# Hypoxic priming of mESCs accelerates vascular-lineage differentiation through HIF1-mediated inverse regulation of Oct4 and VEGF

**DOI:** 10.1002/emmm.201101107

**Published:** 2012-07-23

**Authors:** Sae-Won Lee, Han-Kyul Jeong, Ji-Young Lee, Jimin Yang, Eun Ju Lee, Su-Yeon Kim, Seock-Won Youn, Jaewon Lee, Woo Jean Kim, Kyu-Won Kim, Jeong Mook Lim, Jong-Wan Park, Young-Bae Park, Hyo-Soo Kim

**Affiliations:** 1 Department of Internal Medicine, Innovative Research Institute for Cell Therapy, Seoul National University HospitalSeoul, Korea; 2 National Research Laboratory of Regenerative Sexual Medicine, Department of Urology, Inha University School of MedicineIncheon, Korea; 3 Division of Pharmaceutical Biosciences, Research Institute of Pharmaceutical Sciences, College of Pharmacy, Seoul National UniversitySeoul, Korea; 4 WCU Program, Department of Molecular Medicine and Biopharmaceutical Sciences, Seoul National UniversitySeoul, Korea; 5 WCU Biomodulation Program, Department of Agricultural Biotechnology, Seoul National UniversitySeoul, Korea; 6 Ischemic/Hypoxic Disease Institute, Seoul National University College of MedicineSeoul, Korea

**Keywords:** embryoid bodies, endothelial cells, mesoderm differentiation, mouse embryonic stem cells, niche

## Abstract

Hypoxic microenvironment plays an important role in determining stem cell fates. However, it is controversial to which direction between self-renewal and differentiation the hypoxia drives the stem cells. Here, we investigated whether a short exposure to hypoxia (termed ‘hypoxic-priming’) efficiently directed and promoted mouse embryonic stem cells (mESCs) to differentiate into vascular-lineage. During spontaneous differentiation of embryoid bodies (EBs), hypoxic region was observed inside EB spheroids even under normoxic conditions. Indeed, hypoxia-primed EBs more efficiently differentiated into cells of vascular-lineage, than normoxic EBs did. We found that hypoxia suppressed Oct4 expression via direct binding of HIF-1 to reverse hypoxia-responsive elements (rHREs) in the Oct4 promoter. Furthermore, vascular endothelial growth factor (VEGF) was highly upregulated in hypoxia-primed EBs, which differentiated towards endothelial cells in the absence of exogenous VEGF. Interestingly, this differentiation was abolished by the HIF-1 or VEGF blocking. *In vivo* transplantation of hypoxia-primed EBs into mice ischemic limb elicited enhanced vessel differentiation. Collectively, our findings identify that hypoxia enhanced ESC differentiation by HIF-1-mediated inverse regulation of Oct4 and VEGF, which is a novel pathway to promote vascular-lineage differentiation.

## INTRODUCTION

Embryonic stem cells (ESCs) possess the capacity for self-renewal and pluripotency, and are able to differentiate into many different cell types (Itskovitz-Eldor et al, 2000). However, regulatory mechanisms underlying the differentiation of ESCs into specific cell types are poorly defined, and thus understanding its mechanism might allow us to manipulate the fate of stem cells for stem cell-based therapies. Oxygen concentration, particularly hypoxia (a state of low oxygen), is potentially an important variable for the developing embryo and stem cells. Oxygen gradient and hypoxia exist widely in the developing embryonic tissues, because oxygen diffusion becomes limited owing to the increasing size of the embryo and dense organ structure formation (Semenza, 1999). The oxygen gradient across the growing embryo plays a crucial role in the vascular development in several tissues and organs (Lee et al, 2003; Maltepe & Simon, 1998). Thus, fluctuations in oxygen tension may play an important role throughout embryonic development and in vasculature formation.

Most stem cells exist in complex microenvironments, termed niches (Mohyeldin et al, 2010); stromal cell contacts, extracellular matrix proteins, temperature and low oxygen levels can influence stem cell function and differentiation. Hypoxic microenvironment, in particular, may be a key factor for stem cell phenotypes that lie between the self-renewal and differentiation fates; however, the effects of low oxygen on ESC fates remain controversial and poorly understood. Some studies have shown that hypoxia inhibits differentiation and maintains pluripotency of human ESCs (hESCs) (Ezashi et al, 2005), and improves clonal survival of mouse ESCs (mESCs) (Ying et al, 2008). Cell culture under low oxygen conditions enhances the generation of the induced pluripotent stem cells (iPS cells) of both mouse and human (Yoshida et al, 2009).

In contrast, other studies suggest that low oxygen tension facilitates differentiation. Hypoxia appears to direct the cultured hESCs to differentiate into cardiomyocytes (Niebruegge et al, 2009), or chondrocytes (Koay & Athanasiou, 2008). Hypoxic condition promotes the commitment of ESCs to mesoderm and the generation of hemato-endothelial progenitor cells (Ramirez-Bergeron et al, 2004), and the differentiation to the three germ layers (Powers et al, 2008). In the case of vasculogenic differentiation, low oxygen concentration controls the differentiation potential of human peripheral mononuclear cells, bone marrow CD133^+^ cells, and mESCs (Berthelemy et al, 2009; Ong et al, 2010; Purpura et al, 2008); however, the underlying molecular mechanism remains unclear.

The purpose of this study was to investigate whether low oxygen (1% O_2_) efficiently directs cultured mESCs to differentiate into the vascular lineage, and to elucidate the underlying molecular mechanisms. Given that hypoxia plays an important role in proliferation, differentiation and maintenance of the vascular system during early embryonic development, we hypothesized that exposure of stem cells to hypoxia might facilitate vasculogenic differentiation. We induced spontaneous differentiation of ESCs by forming embryoid bodies (EBs), which is similar to the embryonic gastrulation process (Itskovitz-Eldor et al, 2000), subjected EBs to hypoxia (1% O_2_) for a short duration (so called ‘hypoxic priming of EBs’), and analysed their stem cell fates.

Here, we demonstrated that hypoxic priming enhanced differentiation of EBs into meso-endodermal cells, which differentiated into vascular-lineage cells more efficiently than normoxic EBs did. Particularly with respect to differentiation mechanism, we found for the first time that HIF-1 acts as a suppressor of Oct4 expression through binding to reverse HREs in the Oct4 promoter. Furthermore, hypoxia-primed EBs exhibited higher vascular endothelial growth factor (VEGF) expression, thereby potentiating vascular-lineage commitment. Thus, we conclude that hypoxia promotes ESC differentiation into vascular-lineage through in part by HIF-1−mediated inverse regulation of Oct4 and VEGF, and suggest that hypoxic priming of EBs might be an efficient means to induce ESCs into specific lineages.

## RESULTS

### Hypoxia induces differentiation of mESC-derived EBs to meso-endoderm lineage

**Figure 1:**
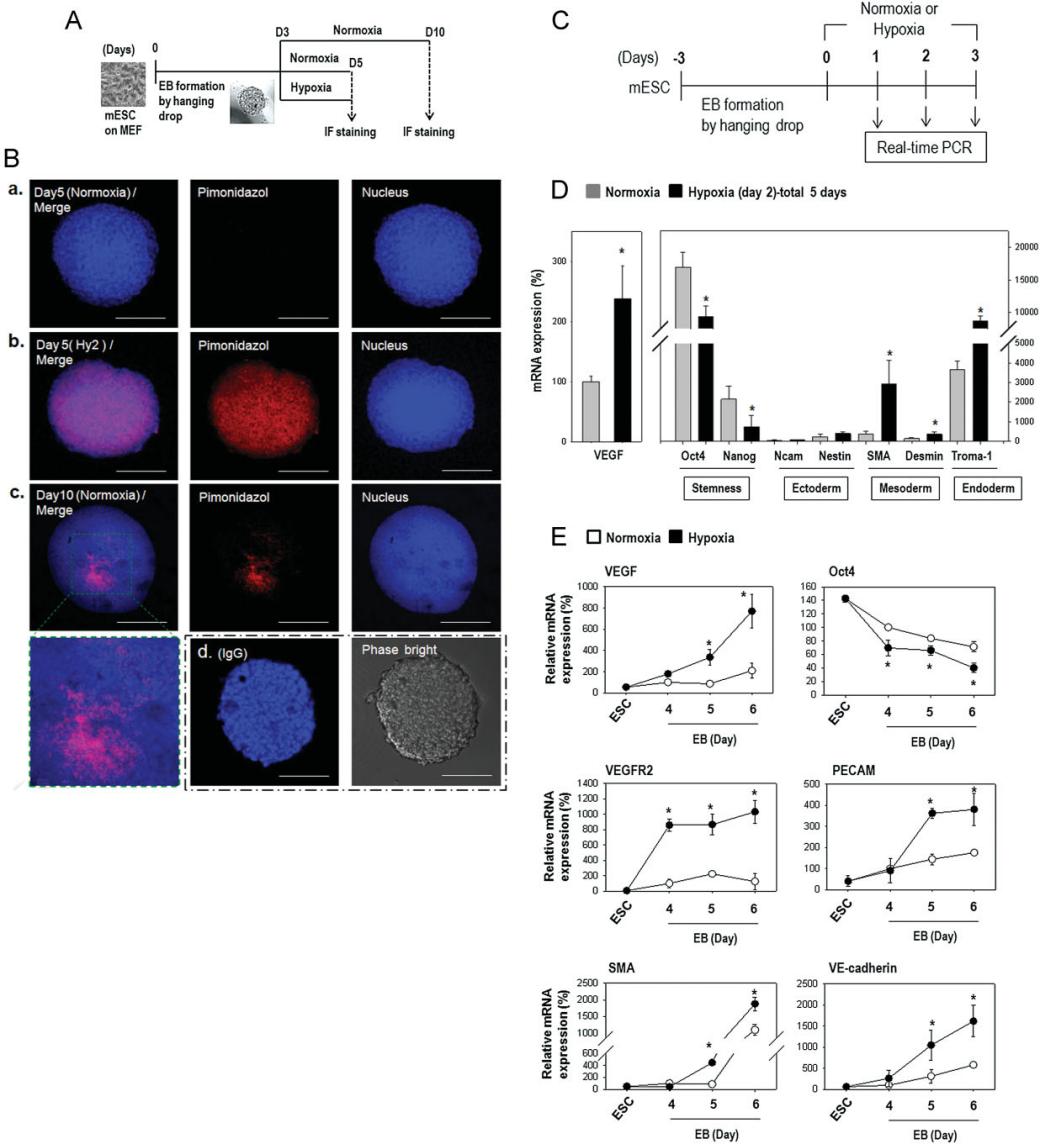
Hypoxia is a natural signal during spontaneous differentiation of ESCs, and hypoxic priming of EBs reduced pluripotency genes whereas induced genes of meso-endoderm and vascular-lineage Embryoid bodies (EBs) were formed using a hanging drop method. The round droplet (500 cells per 20 µl) gathers ESCs and induces cell aggregation (EB). After 3 days, EBs was further incubated in hypoxic or normoxic conditions for immunofluorescence staining.Hypoxic region detected by hypoxic marker, anti-pimonidazole adduct antibody in EBs. EBs was collected on day 5 (a,b) and on day 10 (c). Positive immunoreactivity was detected as red fluorescence (b,c). Negative control treated with mouse isotype-IgG for hypoxia marker in EB. No positive staining was found (d). Magnification = 100×. Scale bar = 100 µM.EBs were made for 3 days in normoxic conditions and then further cultured in normoxia or hypoxic conditions for indicated day(s), respectively. EBs collected for total RNA isolation.EBs made using hanging drop method were exposed to hypoxia for 2 days. The real-time PCR was used to assess the expression of genes associated with pluripotency (Oct4, Nanog), ectoderm (NCAM, Nestin), mesoderm (SMA, Desmin), endoderm (Troma-1) and hypoxia responsive gene (VEGF) (*n* = 4, *p < 0.05).Hypoxia response gene (VEGF) and vascular marker genes (VEGFR2, PECAM, SMA, VE-cadherin) were upregulated in 1% oxygen environments. Oct4 was significantly downregulated compared with normoxia. Graphs show relative percent change over day 4 normoxic-EB (*n* = 4, *p < 0.05).

We examined the effects of hypoxia on the mESC-derived cell aggregates termed EBs. We first determined whether hypoxic regions appeared within EBs during spontaneous differentiation under normoxia (21% O_2_) (Fig 1A and B). EBs were formed using a hanging drop method, and the hypoxic region was measured with the hypoxic marker pimonidazole hydrochloride, which is converted by hypoxia-activated nitroreductases into a reactive intermediate that forms covalent adducts with cellular components in hypoxic regions (Arteel et al, 1995).

Day 5 EBs under hypoxic conditions (1% O_2_), but not normoxic conditions (21% O_2_), exhibited intense staining, indicating the presence of pimonidazole hydrochloride adducts (Fig 1B). Interestingly, positive immunoreactivity for pimonidazole adducts was detectable on day 7 and day 10 in EBs cultured even under normoxic conditions (Fig 1B), supporting our hypothesis that a hypoxic environment might be naturally created inside EB spheroids.

Next, we examined whether culture of EBs in a low oxygen environment affected their commitment to differentiate into a specific lineage. To this end, EBs were exposed to 1% oxygen for up to 3 days (total number of days: 6), and collected at different time points to check the expression of genes marking either pluripotency or differentiation (Fig 1C). At 2 days after hypoxic exposure, we first checked the expression of VEGF, a main downstream factor that is transcriptionally regulated by hypoxia (Semenza, 2003; Fig 1D). VEGF mRNA was more abundant in hypoxic-EBs than in the normoxic control, indicating that the EBs responded to hypoxic stimulus. Interestingly, Oct4 and Nanog, the markers of the undifferentiated state, were significantly downregulated in hypoxic-EBs. Expression of genes marking differentiation towards each embryonic germ layer: ectoderm (NCAM, Nestin), mesoderm (SMA, Desmin) or endoderm (Troma-1) was then monitored. NCAM and Nestin were expressed at very low levels, and their expression was not modulated in response to exposure to hypoxic conditions. In contrast, SMA, Desmin and Troma-1 exhibited remarkable changes, and increased 7.8-, 2.6- and 2.4-fold, respectively, relative to their levels in normoxic-EBs (Fig 1D). These results suggest that hypoxia pushes EBs towards meso-endodermal differentiation.

Because hypoxia during the first few days of differentiation resulted in the preferential differentiation to meso-endoderm in Fig 1D and we are interested in vascular differentiation among mesoderm lineage, we further investigated the expression of vascular differentiation markers (Fig 1E). The hypoxia response gene VEGF was more highly expressed at every time point of hypoxia than that of normoxia (Fig 1E). Oct4 mRNA was remarkably downregulated upon differentiation and its expression was significantly lower at every time point during exposure to hypoxic conditions than during exposure to normoxic conditions. Expression of vascular genes (VEGFR2, PECAM, SMA, VE-cadherin mRNAs) was similarly upregulated during differentiation and was higher in hypoxic-EBs than in normoxic-EBs (Fig 1E). These results suggest that hypoxia-priming of EBs promotes cell differentiation and commits EBs towards vascular-lineage.

### HIF-1α decreases Oct4 transcription through binding to rHREs in the Oct4 promoter in hypoxia

**Figure 2:**
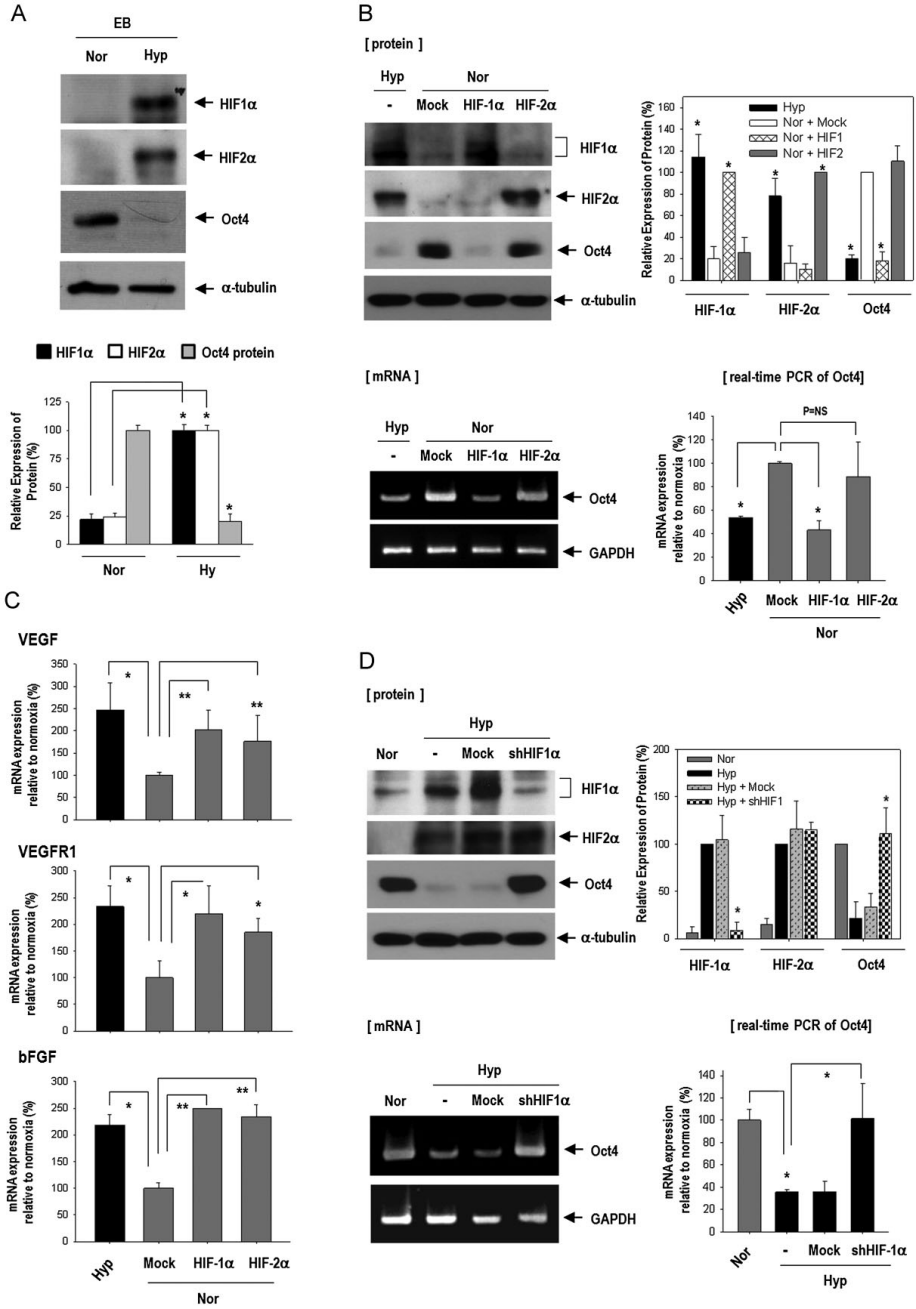
HIF-1α, not HIF-2α, transcriptionally repressed Oct4 EBs formed by hanging drop for 3 days, and cultured under normoxic or hypoxic condition for 16 h. Western blotting of HIF-1α, HIF-2α and Oct4 (left). Quantification graph (right, *n* = 4, *p < 0.05).Western blotting of HIF-1α, HIF-2α and Oct4 for 16 h under 21 and 1% O_2_ (top left). ESCs were transfected with pEGFP-HIF-1α/pEGFP-HIF-1β or pEGFP-HIF-2α/pEGFP-HIF-1β for 24 h and formed EBs. Quantification graph (top right, *n* = 3, *p < 0.05 *versus* Nor). RT-PCR and real-time PCR for Oct4 mRNA (bottom, *n* = 4, *p < 0.001).Quantitative real-time RT-PCR for VEGF, VEGFR1 and bFGF. ESCs were transfected with pEGFP-HIF-1α or pEGFP-HIF-2α for 24 h, formed EBs and further incubated for 16 h under 21 and 1% O_2_ (*n* = 3, *p < 0.01, **p < 0.05).HIF-1α specific knockdown (shHIF1α) significantly reversed the hypoxia-mediated Oct4 reduction in mRNA and protein levels. Western blotting (top, *n* = 3, *p < 0.05 *versus* Hyp), RT-PCR and real-time PCR (bottom, *n* = 4, *p < 0.05).

One of our novel findings is that ESCs were facilitated during hypoxic culture. To determine the mechanism by which hypoxia promoted differentiation, we examined the effect of hypoxia on Oct4 expression. Oct4 is essential for maintaining stem cell self-renewal and pluripotency (Nichols et al, 1998; van den Berg et al, 2010). Levels of Oct4 mRNA (Fig 1) and protein were dramatically reduced by hypoxia (Fig 2A). In contrast, HIF-1α and HIF-2α protein expression exhibited a dramatic increase in response to hypoxia (Fig 2A), supporting the possible involvement of HIF-1α and/or HIF-2α in the down-regulation of Oct4 transcription.

To evaluate the effect of HIF-1α or HIF-2α, we transfected cells with HIF-1α or HIF-2α vector under normoxic conditions (Fig 2B). Both the HIF-1α and HIF-2α groups were co-transfected with their partner HIF-1β (also named ARNT) to enable the formation of a functional transcriptional complex. In Fig 2B, HIF-1α or HIF-2α was highly over-expressed as similar as hypoxic conditions. Interesting point was that Oct4 transcription and protein was significantly decreased by only HIF-1α over-expression (Fig 2B). Expression of hypoxia-regulated genes such as *VEGF*, *VEGFR1* and *bFGF* was increased in both HIF-1α and HIF-2α overexpression groups (Fig 2C), suggesting that the HIF-2α plasmid functioned normally; however, HIF-2α overexpression did not affect Oct4 mRNA and protein levels. To confirm the effect of HIF-1 on Oct4, we knocked down HIF-1α using its shHIF-1α (Fig 2D). By shHIF-1α, HIF-1α protein markedly reduced, whereas control mock vector (Mock) transfection had no effect on HIF-1α protein. In addition, HIF-2α protein was not affected by shHIF-1α. We found that even under hypoxic conditions, HIF-1α inhibition significantly reversed the hypoxia-mediated Oct4 reduction in mRNA and protein levels (Fig 2D).

**Figure 3:**
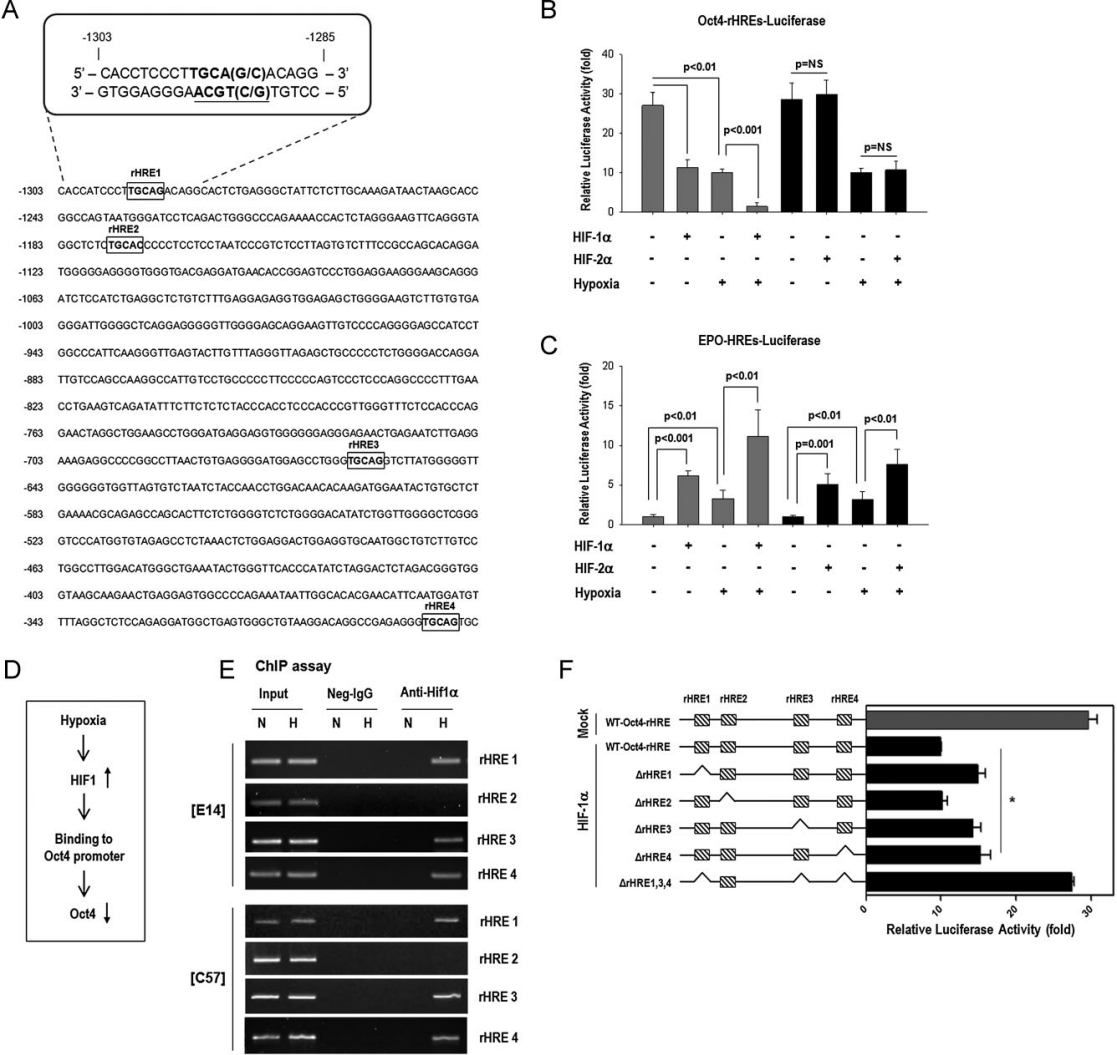
HIF-1α transcriptionally repressed Oct4 by binding to rHREs in the Oct4 promoter under hypoxia **A.** Upstream sequence of the mouse Oct4 promoter (GenBank accession no. S58422) (Nordhoff et al, 2001). Nucleotides are numbered relative to the translation start site, and 4 potential rHREs (5′-TGCA(C/G)-3′) are marked by *squares*.**B,C.** Differentiating E14 ESCs cultured in the absence of LIF and feeder cells were co-transfected with 1 µg pGL3-Oct4-rHRE (**B**), pGL3-EPO-HRE (**C**), 0.2 µg pCMV-β-gal, 0.5 µg pEGFP-HIF-1α or pEGFP-HIF-2α and 0.5 µg pEGFP-HIF-1β plasmid for 24 h and incubated for 16 h under 21 and 1% O_2_ (*n* = 4).**D.** HIF-1 decreases Oct4 expression by binding to rHRE site of Oct4 promoter.**E.** ChIP analysis shows the HIF-1α binding to the Oct4 promoter region. Lysates of differentiating E14- or C57-ESCs exposed to hypoxia for 16 h were immunoprecipitated with antibody for HIF-1α. The precipitated DNAs was evaluated by PCR using specific primers for rHREs (rHRE1 to rHRE4) on Oct4 promoter (*n* = 3 each).**F.** HIF-1α binds to three of four rHRE HIF-binding sites. Schematic diagram of the four rHRE HIF-binding motifs in full-sequence Oct4 promoter and the promoter serial deletion mutants (left). Differentiating E14 ESCs were transfected with various combinations of full-length Oct4 promoter, Oct4 promoter deletion mutants and HIF-1α/β, and promoter luciferase activity was measured at 16 h later (right, *n* = 3 each, *p* < 0.05).

We then evaluated the mechanism by which HIF-1α regulated Oct4 transcription. HIF-1 is a master transcription factor that activates expression of several genes, including VEGF and erythropoietin (EPO), involved in adaptive responses to hypoxia and ischemia by binding to consensus HREs in the gene promoters (Cascio et al, 2008; Keith & Simon, 2007). Previous studies have shown that HIF-1α occasionally acts as a transcriptional repressor of certain genes under hypoxic conditions by direct binding to specific HREs (HRE sequence on the minus DNA strand; Fig 3A). This sequence is known as reverse HRE (rHRE) (Mazure et al, 2002; Narravula & Colgan, 2001). Interestingly, in the mouse Oct4 promoter (Nordhoff et al, 2001) we found four potential rHREs (5′-TGCA(C/G)-3′; Fig 2D) corresponds to HRE sequence for HIF-1 binding (Cascio et al, 2008; Keith & Simon, 2007).

We performed a promoter luciferase assays to determine the effects of HIF-1α or HIF-2α on the Oct4 promoter containing 4 rHREs (Oct4-rHREs; Fig 3B and C). Hypoxia elicited a significant decrease of the reporter gene activity in cells transfected with Oct4-rHREs. HIF-1α also decreased luciferase activity under normoxic or hypoxic conditions (Fig 3B), whereas HIF-2α did not affect the promoter activity of Oct4-rHREs, supporting the involvement of HIF-1α in Oct4 promoter repression through binding to rHREs. As a control, we performed a parallel experiment using EPO reporter vector containing conventional HREs (EPO-HREs), because EPO transcription has been shown to be upregulated by HIF-1 (Semenza, 2003) or HIF-2 (Warnecke et al, 2004). EPO-HRE reporter gene activity was significantly upregulated in hypoxic cells and in cells transfected with HIF-1α or HIF-2α (Fig 3C). These results suggest that HIF-2α plasmid functioned normally; however only HIF-1α decreased Oct4 promoter activity by binding to Oct4-rHREs (Fig 3D).

From these data, we performed ChIP assay to examine the binding of HIF-1α to the four putative rHREs in the Oct4 promoter (Fig 3E). In E14 and C57 mESCs, three of four putative rHREs functioned as binding sites for HIF-1α; however, the second rHRE site (rHRE2) did not (Fig 3E). We confirmed HIF-1α binding to rHREs with serial deletion mutants of the Oct4 promoter (Fig 3F). By HIF-1α overexpression, the luciferase activity was significantly decreased in cells transfected WT-Oct4-rHRE. Interestingly, cell extracts transfected with the ΔrHRE2 plasmid, in which the second rHRE sequence (rHRE2) is deleted, exhibited a remarkable reduction in luciferase activity similar to that elicited by WT-Oct4-rHRE. In contrast, suppression of the Oct4 reporter activity by HIF-1α was partially inhibited in cells transfected with ΔrHRE1, ΔrHRE3 or ΔrHRE4. However, the suppression was almost completely inhibited in cells with combinational rHRE deletion plasmid ΔrHRE1,3,4, indicating that repression of Oct4 promoter activity by HIF-1α largely depends on rHRE1, rHRE3 and rHRE4 sequences, and HIF-1α does not bind to the rHRE2 site, which is consistent with our ChIP data.

### Hypoxia-primed EBs efficiently differentiate into vascular-lineage

**Figure 4:**
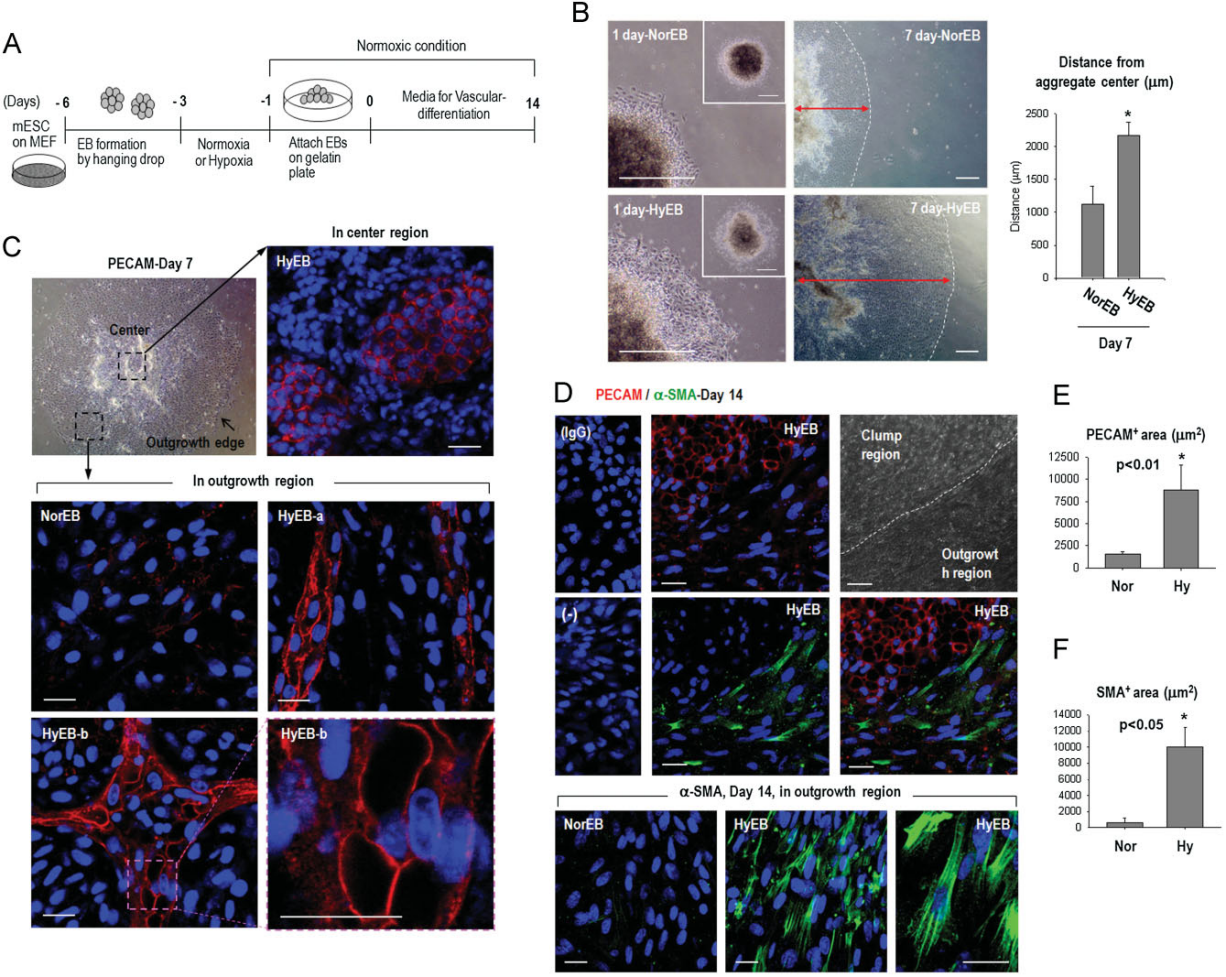
Vascular differentiation is enhanced in hypoxia-primed EBs **A.** EBs formed by hanging drop was cultured under normoxic or hypoxic condition for 2 days, respectively, and attached onto 0.3% gelatin-coated 24-well plate (2–3 EBs per well) in DMEM medium with 10% FBS. On the next day, the culture medium was replaced with vascular differentiation medium, and the cells were further incubated up to 14 days.**B.** Morphology of differentiated EBs on 1 day and 7 days after reattachment. Cells spreading to the surroundings of the reattached aggregates (left and center) showed the appearance of differentiated cells, and the hypoxia-primed EBs (HyEB) spread out far more than normoxic-EBs (NorEB). Quantitative graph of migrating distance from aggregate center on 7 days after reattachment (right). Measurement of three-different points per one EB and 10-EBs were used (*p* < 0.05). Magnification = 40×, scale bar = 300 µm.**C,D.** Immunofluorescence staining against PECAM (red) on day 7 (**C**) and PECAM (red)/α-SMA (green) on day 14 after EB reattachment (**D**). PECAM and α-SMA were very strong in hypoxia-primed EBs. Nucleus for SytoxBlue (blue). NorEB, normoxic-EBs; HyEB, hypoxia-primed EBs. Representative confocal microscopic photographs were shown and no fluorescence signal in isotype IgG control and in negative control. Scale bar = 30 µm. Magnification = 400×.**E,F.** Quantitative graph of PECAM^+^ area (**E**) and SMA^+^ area (**F**) using ImageJ program.

Hypoxic priming of EBs promoted differentiation and enhanced expression of vascular-lineage markers; therefore, we further examined the potential of hypoxia-primed EBs for differentiating into endothelial and smooth muscle cells. EBs were cultured under normoxic or hypoxic condition for 2 days, and plated on 24-well plates coated with 0.3% gelatin. On the next day, the culture medium was replaced with vascular differentiation medium and cells were further incubated up to 14 days (Fig 4A).

The EBs spread out as early as 24 h after attachment to a gelatin-coated plate. Cells spreading to the surroundings of the reattached EBs showed the appearance of differentiated cells. Notably, hypoxia-primed EBs (HyEB) spread out far more than normoxic ones (Fig 4B). We evaluated the vascular (endothelial and smooth muscle cell) differentiation potential of hypoxia-primed EBs by FACS analysis for PECAM, VE-cadherin and SMA on day 3 after reattachment (Supporting Information Fig S1). The fraction of hypoxia-primed EBs (Hy) expressing the endothelial marker PECAM or VE-cadherin, was significantly greater than that of normoxic-EBs (Nor). The percentage of cells expressing SMA, a smooth muscle marker, was similar or not significantly different in the normoxia and hypoxia groups.

We confirmed the differentiation potential towards vascular lineage by staining with anti-PECAM and anti-SMA antibodies (Fig 4C–F). On day 3 after reattachment, PECAM was very rarely stained in hypoxia-primed EBs (HyEB) while it was not detected in normoxic ones (NorEB) (data not shown). PECAM staining (red) in HyEBs increased with time, and showed strong junctional distribution by day 7 after reattachment (Fig 4C). Interestingly, the PECAM^+^ staining differed at different locations. In the centre clump, PECAM^+^ cells had small nuclei and gathered in the honeycomb or cobblestone pattern (Fig 4C; HyEB). In the outgrowth region, the PECAM^+^ cells nuclei were larger than those in the centre clump. PECAM^+^ cells formed tubular structures (HyEB-a) and branching (HyEB-b), indicating that HyEBs efficiently differentiated into endothelial cells. In normoxic EBs (NorEB), however, PECAM^+^ cells were hardly detectable in the centre clump, and those with very weak staining were observed in the outgrowth region. Even detected, PECAM staining was dot-like or punctuate, but not junctional (Fig 4C; NorEB), indicating incomplete differentiation into endothelial cells. SMA and VE-cadherin immunofluorescences were not detectable on day 7 after reattachment in NorEB or HyEB.

On day 14 after reattachment (Fig 4D), strong PECAM staining was detected in the centre clump of HyEB. In the outgrowth region, PECAM staining was weaker than in the centre clump, while SMA^+^ staining was strongly detectable in the outgrowth region of HyEB (Fig 4D). VE-cadherin was also high in the outgrowth region of HyEB on day 14 (data not shown). From these data, we probably made inferences that hypoxia-primed EBs differentiated into vascular-lineage more efficiently than normoxic-EBs did. Furthermore, high hierarchy-vascular cells (PECAM^+^) may be located in the centre clump region of the EBs, and these cells may differentiate into low hierarchy-vascular cells (SMA^+^, VE-cadherin^+^) and move radially.

### HIF-1-mediated VEGF expression is important for hypoxia-primed EB differentiation into vascular-lineage

Because hypoxic priming of EBs enhanced differentiation into vascular-lineage, we further investigated whether HIF-1α was involved in the expression of vascular-lineage markers (Supporting Information Fig S2). To this end, we treated EBs with the HIF-1 blocker YC1 (Yeo et al, 2003) under hypoxic conditions.

**Figure 5:**
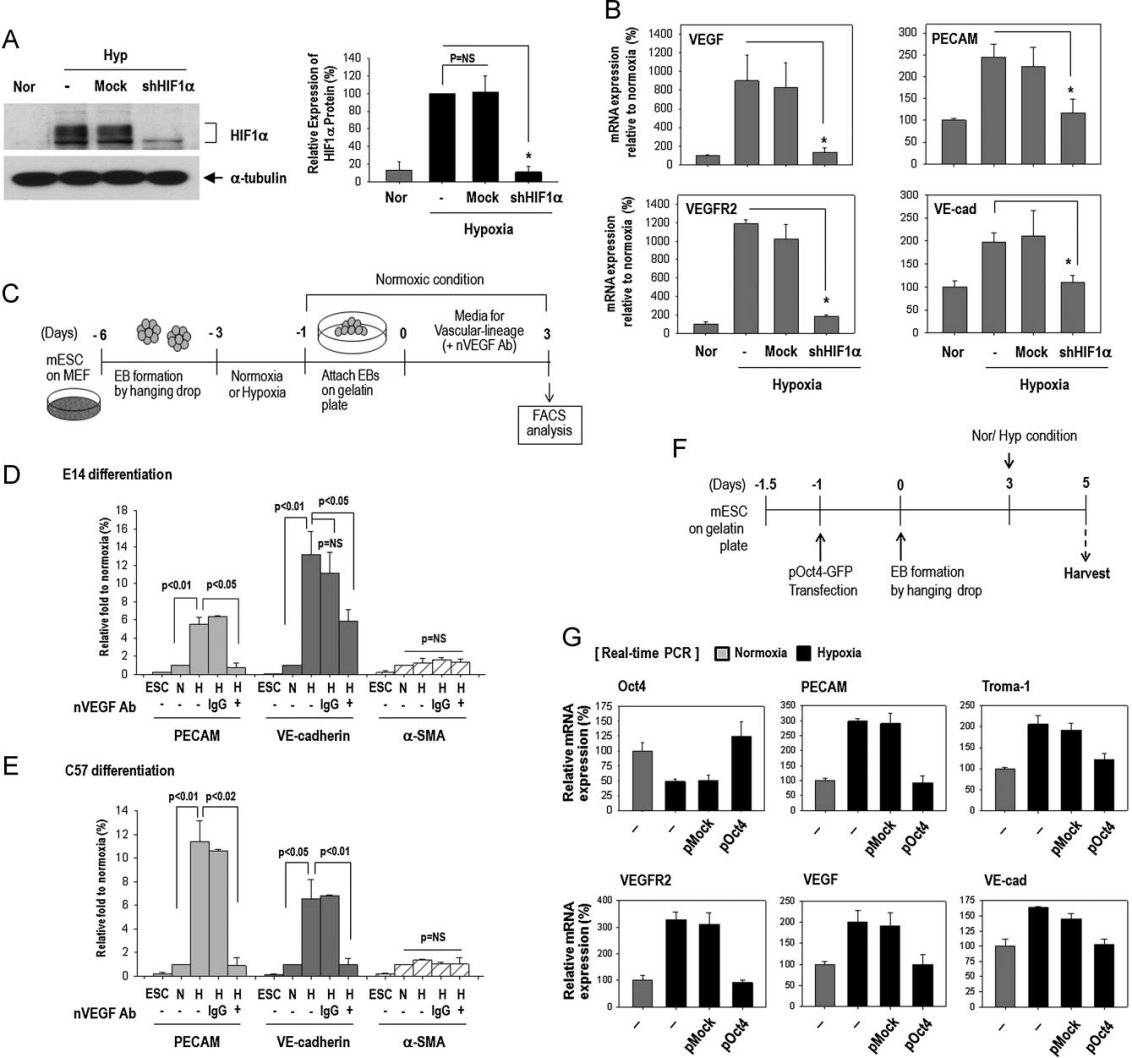
The endothelial differentiation of hypoxia-primed EBs is dependent on HIF-1α and VEGF **A.** HIF-1α protein markedly reduced after the transfection of shHIF-1α. ESCs, Mock or shHIF-1α stable transfectant formed EBs and incubated for 16 h under normoxic or hypoxic conditions. Western blotting of HIF-1α (left). Quantitative graph (right) (*n* = 4, *p < 0.05).**B.** Hypoxia responsive gene (VEGF) and vascular marker genes (PECAM, VEGFR2, VE-cadherin) were investigated with real-time PCR (*n* = 4, *p < 0.05).**C.** EBs were cultured under normoxic or hypoxic condition for 2 days, respectively, and attached onto 0.3% gelatin-coated 24-well plate (2–3 EBs per well) in DMEM medium with 10% FBS. On the next day, the culture medium was replaced with vascular differentiation EGM medium and the cells were further incubated for 3 days. Neutralizing antibody against mouse VEGF (50 µg/ml) or control istotype IgG antibody was added to the culture medium every day.**D,E.** E14 or C57 cells were analysed by FACS for PECAM, VE-cadherin (endothelial cells) and α-SMA (smooth muscle cells) on day 3 after EB reattachment. N, normoxic-EBs; H, hypoxia-primed EBs; nVEGF Ab, neutralizing anti-mVEGF antibody; IgG, istotype IgG antibody. Quantitative graph of FACS analysis from four-different experiments was shown. The percentage was normalized to normoxic group and shown as percent fold (*n* = 4 each).**F,G.** E14 ESCs were transfected with Mock or Oct4 plasmid for 24 h, formed EBs and incubated for 2 days under normoxic or hypoxic conditions. Oct4, VEGF, mesoderm (vasculogenic marker genes; PECAM, VEGFR2, VE-cadherin) and endoderm gene (Troma-1) were investigated with real-time PCR (*n* = 3).

HIF-1α protein level increased in EBs under hypoxia in the absence of YC1, but decreased in a dose-dependent manner in EBs cultured with YC1 (Supporting Information Fig S2a). Further, mRNA expression of VEGF, an HIF-1-responsive gene, showed a dose-dependent decrease in cells cultured with YC1 (Supporting Information Fig S2b). Interestingly, expression of vascular-lineage genes (PECAM, VEGFR2, VE-cadherin) also dose-dependently decreased with increasing concentrations of YC1 (Supporting Information Fig S2b). To support the role of HIF-1 in the regulation of vascular-lineage genes in EBs, we specifically knocked down the expression of HIF-1α (Fig 5). HIF-1α protein markedly reduced after the transfection of shHIF-1α (Fig 5A). Interestingly, expression of VEGF and vascular-lineage genes (PECAM, VEGFR2, VE-cadherin) also decreased in shHIF-1α (Fig 5B). This observation was confirmed with FACS analysis (Supporting Information Fig S3). Hypoxic priming accelerated the endothelial lineage differentiation of EBs. Interestingly, shHIF1/Hy showed a remarkable reduction of this differentiation effect. These observations imply that HIF-1 may play an important role in vascular differentiation *in vitro*.

We further focused on the role of VEGF in endothelial cell differentiation in both E14 and C57 mESCs (Fig 5C–E), because VEGF is a well-established endothelial growth and differentiation factor (Olsson et al, 2006). The addition of exogenous VEGF is usually required to induce stem cells to differentiate into endothelial cells (Nourse et al, 2010; Vittet et al, 1996). We found that hypoxic priming accelerated the endothelial lineage differentiation of EBs without additional exogenous VEGF (Fig 5C–E and Supporting Information Fig S1), which is mainly driven by the endogenous VEGF that is highly produced in hypoxia-primed EBs (Figs 1 and 5B). Using ELISA, we confirmed VEGF secretion from hypoxia-primed EBs; the VEGF levels were sustained during the subsequent differentiation period (data not shown). The cells expressing endothelial marker (PECAM or VE-cadherin) in hypoxia-primed EBs (Hy) was significantly higher than in normoxic-EBs (Nor), which was reversed by addition of neutralizing antibody against VEGF (Hy + nVEGF) (Fig 5D and E). These findings suggest that hypoxia stimulates EBs to produce VEGF, which, in turn, may activate endothelial differentiation in an autocrine and/or paracrine manner.

We also wondered the role of Oct4 upregulation in vascular differentiation (Fig 5F and G). We overexpressed Oct4, generated EBs, performed hypoxic priming, and examined the expression of several genes with real-time PCR (Fig 5F). Oct4 mRNA expression was strongly upregulated in Oct4-transfected cells even after hypoxic priming. Expression of vascular differentiation markers (PECAM, VE-cadherin and VEGFR2) among mesoderm lineage was upregulated in hypoxia priming group, but was suppressed in Oct4-transfected cells. Expression of the endodermal gene Troma-1, which was elevated by hypoxia, was also suppressed in Oct4-transfected cells even after hypoxic priming (Fig 5G). Unexpectedly, the expression of VEGF was also suppressed in Oct4-transfected cells. These results suggest that re-expression of self-renewal genes, such as Oct4, may inhibit stem cell differentiation.

### Hypoxic priming enhances EB differentiation into endothelial cells *in vivo*

**Figure 6:**
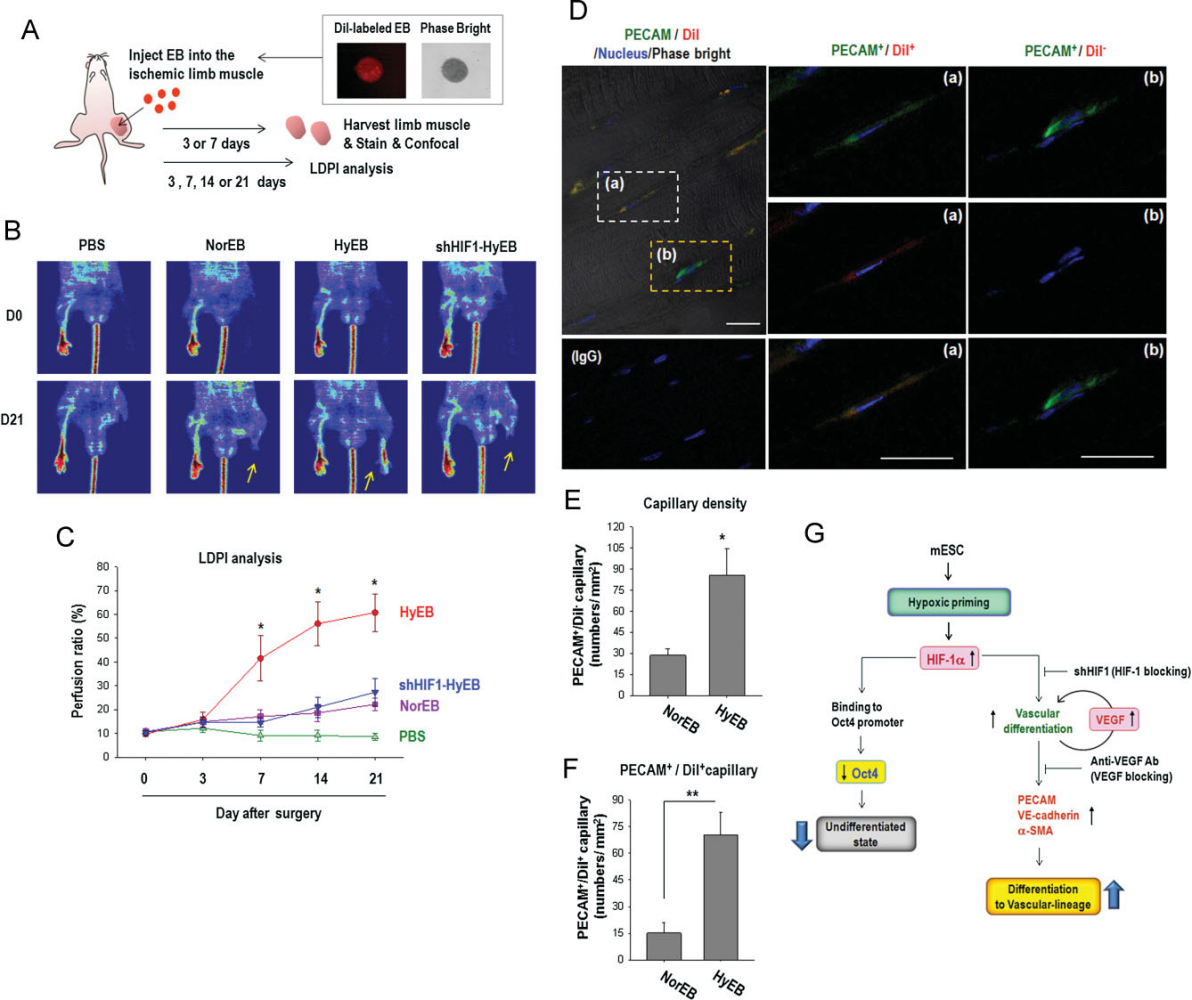
*In vivo* vasculogenic potential of hypoxia-primed EBs is confirmed in mice hind limb ischemic model **A.** BALB/c-nude mice underwent hindlimb ischemic surgery, and then normoxic-EBs (NorEB), hypoxia-primed EBs (HyEB) or hypoxia-primed shHIF1-EBs (shHIF1-HyEB) was intramuscularly injected. To trace differentiating EBs, E14 cells were labelled with DiI (red) before EB formation.**B.** LDPI images. In the HyEB-injection group, the blood flow of ischemic limbs was recovered (arrow).**C.** LDPI was sequentially evaluated after transplantation of each EBs into hindlimb ischemic BALB/c-nude mice (*n* = 5 each, *p* < 0.01).**D.** The adductor muscle on day 7 post-transplantation was longitudinally sectioned and analysed by confocal microscopy for PECAM (green). Capillary endothelial cells stained with PECAM are detected adjacent to skeletal myocytes (phase bright) and are co-localized with DiI immunofluorescence (red). No fluorescence signal in the IgG control group. Magnification = 400×, scale bar = 20 µm.**E,F.** Number of DiI^−^/PECAM^+^ capillaries (**E**) or DiI^+^/PECAM^+^ cells (**F**) incorporating into ischemic limb muscle were presented (numbers/mm^2^, *n* = 5 each, *p < 0.001, **p < 0.05).**G.** Proposed model for enhancement of vascular differentiation of hypoxia-primed differentiating ESCs.

Next, we examined whether hypoxia-primed EBs underwent vascular differentiation in ischemic tissues *in vivo* (Fig 6). We removed the femoral artery of BALB/c-nude mice and transplanted normoxic-EBs (NorEB), hypoxia-primed EBs (HyEB) or hypoxia-primed shHIF1-EBs (shHIF1-HyEB) into the adductor muscle (Fig 6A). The differentiated cells from DiI-labelled EBs express red DiI-fluorescence and are easily detectable for tracking and quantification.

The NorEB-injection group exhibited progressive limb loss; on the other hand, ischemic limbs in the HyEB-injection group were in a comparatively good condition (Supporting Information Fig S4). In Fig 6B and C, the PBS group without cell injection showed no blood flow recovery, whereas injection with NorEB slightly restored hindlimb blood flow. Injection of HyEB significantly restored hindlimb blood flow. Notably, injection group of shHIF1-HyEB resulted in a significantly reduced recovery of hindlimb blood flow (Fig 6B and C), supporting our hypothesis that hypoxic priming enhances vascular-lineage differentiation and HIF-1 may play an important role in this differentiation. In the histological analysis also, we observed many capillary structures in the HyEB-injected mouse limb. As shown in Fig 6D, capillaries stained with PECAM (PECAM^+^) were detected adjacent to skeletal myocytes (phase bright), which was more frequently found in HyEB group than in NorEB one (Fig 6E and F). Interestingly we found a mass in the injected limb as well as metastatic masses in the liver of the NorEB-injection group, but not the HyEB-injection group, suggesting that hypoxia-primed EBs are at the more differentiated status than normoxic-EBs are.

Capillaries stained with VE-cadherin (white) and BS1-lectin (green), another endothelial marker, are also co-localized with DiI immunofluorescence (red) (Supporting Information Fig S5), indicating that transplanted EBs undergo vasculogenic differentiation *in vivo*, and that hypoxia-primed EBs differentiate into vessels more efficiently than normoxic-EBs do.

## DISCUSSION

A major new finding of the present study is that hypoxia can regulate ESCs' differentiation status and direction via HIF-1-mediated control of both Oct4 and VEGF expression. We demonstrate here that HIF-1 is a direct upstream negative regulator of Oct4, a key factor for maintaining stem cell pluripotency, whereas HIF-1 increases the expression of VEGF, an endothelial growth and differentiation factor. Hypoxic priming facilitates ESCs to differentiation status and engages them towards the vascular-lineage (Fig 6G).

### Hypoxia in stem cell niches: maintenance of stemness *versus* differentiation?

Stem cell ‘niches’ are complex microenvironments with physical, chemical and biological cues, and have been implicated as directors of stem cell fate (Ohlstein et al, 2004). Low oxygen microenvironment (hypoxic niche) is one of the important ‘niche’ components in determining the fate of stem cells (Davy & Allsopp, 2011). It is controversial to which direction between pluripotency and differentiation the low oxygen level drives stem cells.

Several reports have suggested that low oxygen or HIF promotes the undifferentiated state of human and mouse ESCs (Ezashi et al, 2005; Ying et al, 2008). Hypoxia promotes a stem cell-like phenotype of the non-stem cell population in glioblastoma via upregulation of Oct4, Nanog and c-Myc (Heddleston et al, 2009). Hypoxia alters gene expression and promotes an immature and neural crest progenitor phenotype in human neuroblastoma cells via HIF-1 and HIF-2 (Jogi et al, 2002). Activation of the Notch signalling pathway by HIF-1 maintains the undifferentiated state and thus enhances the generation of both human and mouse iPS cells (Yoshida et al, 2009).

Conversely, hypoxia or HIFs potentiates stem cell differentiation. Culture of neuroprogenitors at hypoxic conditions promotes neuronal differentiation (Francis & Wei, 2010), and knock-down of HIF-1α decreases the neuron differentiation (Tomita et al, 2003). Hypoxia enhances the differentiation of hESC and mESC towards cardiomyocytes (Niebruegge et al, 2009). HIF-1α knock-out mESCs do not produce spontaneously beating cells (Ateghang et al, 2006), but exogenous expression of HIF-1α promotes cardiac differentiation of mESCs (Ng et al, 2010). HIF-2α can function as a tumour suppressor in rat glioma xenografts (Acker et al, 2005), suggesting that HIF-2α decreases the stem-like phenotype of the stem and non-stem populations in glioma.

Such a great controversy on the effect of hypoxia on the stemness may be derived from various factors; the hierarchy of stemness when exposed to hypoxia, duration of hypoxic exposure, balance between inducer and inhibitor of differentiation, cell type specific differences in signalling pathways, and so on. In our study, hypoxia potentiated ESC differentiation towards endo-mesodermal lineage (Fig 1). Thus we believe at least this specific situation that exposure of stem cells (at the early stage of spontaneous differentiation ESCs including EBs) to hypoxia for a couple of days induces the stem cells to differentiate into meso-endodermal lineage. The truth of this notion is well corroborated by the findings that HIF-1 binds to rHREs in Oct4 promoter region and suppresses its expression (Figs 2 and 3).

### Mechanism of hypoxia to facilitate differentiation; HIF-1α suppresses Oct4

For the maintenance of stem cell pluripotency and stemness, several regulators such as Oct4, Nanog and Sox2 are known to be important (Boyer et al, 2005). Especially, the POU transcription factor Oct4 (also known as Oct3/4 and Pou5F1) has been known as a master regulator of pluripotency (Boyer et al, 2005). Oct4 plays a critical role in regulating ESC differentiation and maintaining the pluripotent nature of ESCs and primordial germ cells (Nichols et al, 1998; van den Berg et al, 2010). Reduction in Oct4 expression causes ESCs to lose pluripotency and differentiate into trophectoderm, mesoderm, neuroectoderm or endoderm lineages (Niwa et al, 2000). Ectopic Oct4 expression blocks progenitor cell differentiation and also contributes to tumour growth (Gidekel et al, 2003; Hochedlinger et al, 2005). Therefore, Oct4 regulation might be important in determination of stem cell fate. But mechanisms and factors regulating Oct4 expression have not been fully elucidated.

In our study, we found that HIF-1α bound the Oct4 promoter and decreased Oct4 expression (Figs 2 and 3). Oct4 downregulation by HIF-1α provides a novel mechanism to explain the effective induction of stem cell differentiation by hypoxia and HIF-1α.

Covello and coworkers (Covello et al, 2006) reported a little bit different results from ours. In their study with HIF-2α knock-in mice, Oct4 expression was upregulated; thus, they suggested that HIF-2α regulates stem cell function by inducing Oct4 overexpression in HIF-2α knock-in mice and in primordial germ cells. But, this Oct4 inducing effect appears to be specific for HIF-2α, because HIF-1α does not induce Oct4 in renal carcinoma cells (Covello et al, 2006).

In contrast to this previous observation that HIF-1α does not bind to the Oct4 promoter in renal carcinoma cells (Covello et al, 2006), our data show that HIF-1α decreases Oct4 expression and indeed binds to the Oct4 promoter, which was confirmed in two different mESC lines, E14 and C57. Moreover, we observed that Oct4 transcription and protein was decreased by only HIF-1α overexpression, and HIF-1α knockdown significantly reversed the hypoxia-mediated Oct4 reduction in mRNA and protein levels even under hypoxic conditions. The reasons for the discrepancy between our data on Oct4 regulation by HIFs and the findings of the other groups are not clear; cell type differences may be a contributing factor. We used differentiating ESCs, whereas Covello's group used primordial germ cells, renal cell carcinoma, and an *in vivo* knock-in system. Further work will be required to clarify this issue.

### VEGF: a mechanism to induce ESCs to vascular-lineage by hypoxia

Embryonic stem cells can differentiate into a wide spectrum of cell types such as cardiomyocytes, endothelial cells and hematopoietic cells (Itskovitz-Eldor et al, 2000). These lineages arise from distinct mesoderm subpopulations that develop sequentially from pre-mesodermal cells (Kouskoff et al, 2005). Hypoxia-exposed EBs showed enhanced differentiation into meso-endodermal lineage, and further differentiated into vascular lineage (Figs 1 and 4, and Supporting Information Fig S3). Interesting point is that treatment of VEGF-neutralizing antibody to hypoxia-primed EBs during differentiation remarkably decreased vascular-lineage differentiation (Fig 5C–E).

Vascular endothelial growth factor is absolutely required for vascular formation during embryonic development, as VEGF-null mice die early in development because of a lack of blood vessels (Carmeliet et al, 1996). For therapeutic vascularization and tissue engineering, an appropriate source of endothelial cells is required; however it remains inefficient to get endothelial cells from ESCs. Therefore, an angiogenic or a growth factor was added to enhance endothelial differentiation. VEGF addition successfully induces the differentiation of endothelium from mouse EBs (Vittet et al, 1996), human ESCs (Nourse et al, 2010) and human breast tumour stem cells (Bussolati et al, 2009). VEGF and bFGF treatments induce endothelial differentiation of mouse MSCs (Wang et al, 2010). However, in our study, we did not add VEGF in EBs' culture. Only hypoxic priming successfully and efficiently induced EBs to differentiate into vascular-lineage cells. Neutralizing antibody against VEGF remarkably blocked the endothelial differentiation of hypoxia-primed EBs (Figs 4 and 5). Thus, we believe that hypoxia-primed EBs produce VEGF which might turn on endothelial differentiation in an autocrine and/or paracrine manner. Such a source of endothelial cells differentiated from ESCs might be advantageous, because genetic modification and exogenous growth factor treatment are not required to achieve endothelial differentiation.

In addition, we observed the existence of hypoxic area in spontaneously differentiating EBs (Fig 1B). VEGF expression increased in a time-dependent manner in spontaneously differentiating EBs, and was indeed upregulated by hypoxic treatment of EBs (Fig 1D and E). HIF-1 is a well-known transcription factor for VEGF expression in response to hypoxia (Semenza, 2003) and plays an important role in vasculature development. Knockout mutations of HIF-1α (Ryan et al, 1998) and HIF-1β (ARNT) (Maltepe et al, 1997) lead to gestational lethality of murine embryos. Phenotypes of these knockouts include reduction in vasculature, blood cells and heart formation. These findings suggest that proper responses to hypoxia are vital for proper vascular formation. When we blocked the HIF-1α, VEGF expression was decreased and resulted in reduction of endothelial cell markers PECAM, VEGFR2 and VE-cadherin in hypoxia-primed EBs (Fig 5 and Supporting Information Fig S3). Consequently, we suggest that HIF-1α-mediated VEGF upregulation is an important determinant of vascular-lineage commitment in hypoxia-primed EBs.

In conclusion, the present data collectively provide insights into the mechanisms by which hypoxia induces ESC differentiation at early stage via suppression of Oct4. Moreover, we provide evidence to support that HIF-1 acts as a negative regulator of Oct4 expression, whereas HIF-1 increased the expression of VEGF, thereby facilitate vascular differentiation. Therefore, we established a potential link between vascular differentiation of ESCs and low oxygen tension. Hypoxic priming of EBs is a good approach to promote endothelial differentiation of ESCs.

## MATERIALS AND METHODS

### Stem cell culture and *in vitro* differentiation

Undifferentiated E14 mESCs and the C57BL/6-background mESCs (C57-mESCs, accession no. SCRC-1002; ATCC) were cultured on Mitomycin C (Sigma–Aldrich)-treated mouse embryonic fibroblast (MEF) feeder layer in Dulbecco's modified Eagle's medium (DMEM; GIBCO, Grand Island, NY, USA) supplemented with 10% FBS (Hyclone), 1% penicillin/streptomycin (GIBCO), 0.1 mM β-mercaptoethanol (Sigma), 1% non-essential amino acids (GIBCO), 2 mM l-glutamine and 1000 U/ml leukaemia inhibitory factor (LIF; Millipore). To induce differentiation, ESCs were cultured in the absence of LIF and feeder cells or EBs were formed using a hanging drop method in the absence of LIF and feeder cells. After 3 days, EBs were further incubated in normoxic or hypoxic conditions for spontaneous differentiation. HIF-1α knock-down E14 cells were obtained using stable transfection with shHIF-1α vector followed by puromycine selection (10 µg/ml: Sigma). To assess *in vitro* vascular differentiation, EBs from hypoxic or normoxic incubators for 2 days were plated and grown onto 0.3% gelatin-coated 24-well plate in DMEM/10% FBS. One day later, culture medium was replaced with EGM2 medium (Lonza) supplemented with 5% FBS and cultured for another 3, 7 or 14 days for vascular differentiation.

### Hypoxic condition and hypoxia marker

For hypoxic conditions, cells were incubated in a Hypoxia Chamber (Forma Scientific) maintaining low oxygen tension (1% O_2_, 5% CO_2_ and balanced with N_2_). Forma Hypoxia Chamber (anaerobic system) is more strict control of hypoxia with a closed hypoxia workstation. Hypoxia marker, pimonidazole hydrochloride and mouse monoclonal antibody for pimonidazole adduct were supplied by Hypoxyprobe™, Inc. Pimonidazole hydrochloride was added in culture media 1 h before EB collection, and then EBs were fixed in 4% PFA for immunofluorescence staining.

### Mouse hindlimb ischemia model and laser Doppler perfusion imaging analysis

All animal experiments performed under approval from the Institutional Animal Care and Use Committee of Seoul National University Hospital. Male BALB/c-nude (8 weeks old) mice were anaesthetized, the unilateral femoral artery was ligated and removed. EBs (total cell numbers corresponds to 5 × 10^4^/100 µl PBS) were intramuscularly injected. To trace differentiated cells, E14 EBs were labelled with 2 µg/ml of DiI (Molecular Probes, Inc.) for 30 min before EB formation by hanging drop. To evaluate the EB differentiation into endothelial cells, the adductor muscles were harvested for immunofluorescence staining. For evaluating the therapeutic blood flow recovery, we used a laser Doppler perfusion imaging (LDPI) analyser (Moor Instrument) as previously described (Lee et al, 2009).

**The paper explained**

PROBLEM:

Embryonic stem cells (ESCs) possess the capacity for self-renewal and pluripotency, and are able to differentiate into many different cell types. However, regulatory mechanisms underlying the differentiation of ESCs into specific cell types are poorly defined, and thus understanding its mechanism might allow us to manipulate the fate of stem cells for stem cell-based therapies.

Hypoxic microenvironment plays an important role in the proper embryonic development, especially in vasculature formation. Also, hypoxia is one of the important ‘niche’ components in determining the fate of stem cells. But the effects of low oxygen levels on stem cell fates (self-renewal and differentiation) are still not clear and controversial.

The aim of this study is to investigate whether low oxygen (1% O_2_) efficiently directs the cultured mouse ESCs to differentiate into the specific lineage, especially the vascular-lineage, and if it does, by which molecular mechanisms.

RESULTS:

We used differentiation cultures that make mESC develop into EBs to mimic early embryonic development, and a short time exposure to hypoxia (termed ‘hypoxic priming’) before inducing vascular-lineage differentiation. Given that hypoxia plays an important role in proliferation, differentiation and maintenance of the vascular system during early embryonic development, we hypothesized that exposure of stem cells to hypoxia might facilitate vasculogenic differentiation. Here, we have shown that hypoxic priming enhanced differentiation of EBs into meso-endodermal cells, which differentiated into vascular-lineage cells more efficiently than normoxic EBs did. *First*, hypoxic priming decreased the pluripotency. The mechanism is that HIF-1 suppressed Oct4, a key factor for maintaining stem cell pluripotency, via binding to reverse hypoxia-responsive elements (rHREs) in the Oct4 promoter. Second, hypoxic priming of EBs highly increased the HIF-1-mediated VEGF, an endothelial growth and differentiation factor, thereby successfully differentiating them to vascular-lineage. Our findings proposed that hypoxic priming of ESCs might be an efficient means of inducing ESCs into specialized lineage such as vascular-commitment.

IMPACT:

A major new finding of the present study is that hypoxia can regulate ESCs' differentiation status. Hypoxic priming efficiently engages ESCs to the vascular-lineage. For therapeutic vascularization and tissue engineering, an appropriate source of endothelial cells is required; however it remains inefficient to get endothelial cells from ESCs. Therefore, an angiogenic or a growth factor such as VEGF was added or genetic manipulation was adopted to enhance endothelial differentiation. However, in our study, we did not add VEGF in EBs' culture. Only hypoxic priming successfully and efficiently induced EBs to differentiate into vascular-lineage cells. Such a source of endothelial cells differentiated from ESCs might be advantageous, because genetic modification and exogenous growth factor treatment are not required to achieve endothelial differentiation.

### Real-time PCR

Total RNA was prepared using QIAshredder and RNeasy mini kit (Qiagen, Inc.) according to the manufacturer's instructions. One microgram of RNA was converted into cDNA according to the PrimeScript™ 1st strand cDNA Synthesis Kit (Takara). PCR was performed using the SYBR Green PCR Master Mix (Roche) with specific primers (Supporting Information Table S1). Real-time samples were run on an ABI PRISM-7500 sequence detection system (Applied Biosystems). GAPDH was simultaneously run as a control and used for normalization.

### Western blot analysis

Cells were harvested and lysed in lysis buffer containing protease inhibitors (Roche). Total protein (10–30 µg) was immunoblotted with primary antibodies against HIF-1α (Yeo et al, 2003), HIF-2α (Novus) and Oct4 (Santa Cruz Biotechnology). α-tubulin (Calbiochem) was used as an internal control. Quantification of band intensity was analysed using TINA 2.0 (RayTest) and normalized to the intensity of α-tubulin.

### Luciferase assay

A partial genomic DNA sequence encompassing the promoter region of Oct4 bearing 4 putative rHREs was obtained by genomic PCR and cloned into an enhancer-less luciferase pGL3 promoter vector (Promega). Differentiating E14- or C57-ESCs cultured in the absence of LIF and feeder cells were plated at a density of 2 × 10^5^ cells per well of a 6-well plate and transfected with various combinations of effector plasmids. After transfection, luciferase assays were performed using the Luciferase Assay System kit (Promega) and a luminometer (Turner Design). The relative luciferase activity was normalized to relative light units/β-galactosidase activity.

### Chromatin immunoprecipitation (ChIP) assay

Chromatin immunoprecipitation was performed with the ChIP assay kit (Upstate) according to the manufacturer's protocol. Anti-HIF-1α antibody (Yeo et al, 2003) used for immunoprecipitation of the DNA fragments. The PCR primer were designed to amplify a region of the Oct4 Promoter (-1303 to -286) harbouring 4 putative rHREs (Supporting Information Table S1).

### Fluorescence-activated cell sorter (FACS) analysis

Vascular differentiation of E14 and C57 cells was quantified using FACS analysis. Differentiated cells were detached with 0.05% Trypsin-EDTA. Single cells (at least 1 × 10^4^) were distributed into FACS tube (BD Falcon) and fixed with 1% PFA on ice for 20 min. For inhibiting VEGF effect, neutralizing antibody against mouse VEGF (R&D) was used. For FACS analysis, cells were incubated with anti-PECAM (Santa Cruz biotechnology), anti-VE-cadherin (Santa Cruz Biotechnology) or anti-SMA (Abcam) and bound secondary anti-mouse Alexa 660 or anti-rabbit Alexa 555 (Molecular Probes). Flow cytometric analysis performed using BD CantoII.

### Immunofluorescence staining

To analyse tissues of hindlimb ischemia mice, the adductor muscle was excised 7 days after transplantation, rinsed with PBS and frozen in liquid nitrogen. Ten micrograms-thick histological sections were prepared from snap-frozen tissue samples, fixed with 4% PFA, blocked in 1% BSA and incubated with anti-PECAM (BD Pharmingen) or anti-VE-cadherin (SantaCruz) followed by Alexa 488 or Alexa 647 secondary antibody (Molecular Probes). For quantification of capillary, at least five randomly selected fields from longitudinal sectioned slides were examined and averaged. Numbers of positive cells are presented as number of cells per square millimetre. EBs or differentiated cells on cover-slips were fixed with 4% PFA, blocked with blocking buffer (0.5% goat serum, 0.1% triton-x100/1% BSA-PBS), and labelled with anti-pimonidazole adduct (Hypoxyprobe™, Inc.), anti-PECAM (BD Pharmingen) or anti-SMA (Abcam) followed by Alexa 488 or Alexa 555 secondary antibodies. The nuclei were stained with Sytox Blue (Molecular probe) and mounted using fluorescent mounting medium (DAKO). The fluorescent image was obtained with a fluorescence microscope (Olympus IX71, Japan) and confocal microscope (Carl Zeiss LSM710).

### Statistical analysis

The results are expressed as means ± standard deviations (SD). The differences between the groups were compared by the unpaired *t*-test or one-way analysis of variance (ANOVA), followed by *post hoc* analysis with the Bonferroni test. *p* Values ≤0.05 were considered statistically significant. All statistical analyses were performed using SPSS 17.0 (SPSS Inc., Chicago, US).

## Supplementary material

Detailed facts of importance to specialist readers are published as ”Supporting Information”. Such documents are peer-reviewed, but not copy-edited or typeset. They are made available as submitted by the authors.

## References

[b1] Acker T, Diez-Juan A, Aragones J, Tjwa M, Brusselmans K, Moons L, Fukumura D, Moreno-Murciano MP, Herbert JM, Burger A (2005). Genetic evidence for a tumor suppressor role of HIF-2alpha. Cancer Cell.

[b2] Arteel GE, Thurman RG, Yates JM, Raleigh JA (1995). Evidence that hypoxia markers detect oxygen gradients in liver: pimonidazole and retrograde perfusion of rat liver. Br J Cancer.

[b3] Ateghang B, Wartenberg M, Gassmann M, Sauer H (2006). Regulation of cardiotrophin-1 expression in mouse embryonic stem cells by HIF-1alpha and intracellular reactive oxygen species. J Cell Sci.

[b4] Berthelemy N, Kerdjoudj H, Schaaf P, Prin-Mathieu C, Lacolley P, Stoltz JF, Voegel JC, Menu P (2009). O2 level controls hematopoietic circulating progenitor cells differentiation into endothelial or smooth muscle cells. PLoS One.

[b5] Boyer LA, Lee TI, Cole MF, Johnstone SE, Levine SS, Zucker JP, Guenther MG, Kumar RM, Murray HL, Jenner RG (2005). Core transcriptional regulatory circuitry in human embryonic stem cells. Cell.

[b6] Bussolati B, Grange C, Sapino A, Camussi G (2009). Endothelial cell differentiation of human breast tumour stem/progenitor cells. J Cell Mol Med.

[b7] Carmeliet P, Ferreira V, Breier G, Pollefeyt S, Kieckens L, Gertsenstein M, Fahrig M, Vandenhoeck A, Harpal K, Eberhardt C (1996). Abnormal blood vessel development and lethality in embryos lacking a single VEGF allele. Nature.

[b8] Cascio S, Bartella V, Auriemma A, Johannes GJ, Russo A, Giordano A, Surmacz E (2008). Mechanism of leptin expression in breast cancer cells: role of hypoxia-inducible factor-1alpha. Oncogene.

[b9] Covello KL, Kehler J, Yu H, Gordan JD, Arsham AM, Hu CJ, Labosky PA, Simon MC, Keith B (2006). HIF-2alpha regulates Oct-4: effects of hypoxia on stem cell function, embryonic development, and tumor growth. Genes Dev.

[b10] Davy P, Allsopp R (2011). Hypoxia: are stem cells in it for the long run. Cell Cycle.

[b11] Ezashi T, Das P, Roberts RM (2005). Low O2 tensions and the prevention of differentiation of hES cells. Proc Natl Acad Sci USA.

[b12] Francis KR, Wei L (2010). Human embryonic stem cell neural differentiation and enhanced cell survival promoted by hypoxic preconditioning. Cell Death Dis.

[b13] Gidekel S, Pizov G, Bergman Y, Pikarsky E (2003). Oct-3/4 is a dose-dependent oncogenic fate determinant. Cancer Cell.

[b14] Heddleston JM, Li Z, McLendon RE, Hjelmeland AB, Rich JN (2009). The hypoxic microenvironment maintains glioblastoma stem cells and promotes reprogramming towards a cancer stem cell phenotype. Cell Cycle.

[b15] Hochedlinger K, Yamada Y, Beard C, Jaenisch R (2005). Ectopic expression of Oct-4 blocks progenitor-cell differentiation and causes dysplasia in epithelial tissues. Cell.

[b16] Itskovitz-Eldor J, Schuldiner M, Karsenti D, Eden A, Yanuka O, Amit M, Soreq H, Benvenisty N (2000). Differentiation of human embryonic stem cells into embryoid bodies compromising the three embryonic germ layers. Mol Med.

[b17] Jogi A, Ora I, Nilsson H, Lindeheim A, Makino Y, Poellinger L, Axelson H, Pahlman S (2002). Hypoxia alters gene expression in human neuroblastoma cells toward an immature and neural crest-like phenotype. Proc Natl Acad Sci USA.

[b18] Keith B, Simon MC (2007). Hypoxia-inducible factors, stem cells, and cancer. Cell.

[b19] Koay EJ, Athanasiou KA (2008). Hypoxic chondrogenic differentiation of human embryonic stem cells enhances cartilage protein synthesis and biomechanical functionality. Osteoarthritis Cartilage.

[b20] Kouskoff V, Lacaud G, Schwantz S, Fehling HJ, Keller G (2005). Sequential development of hematopoietic and cardiac mesoderm during embryonic stem cell differentiation. Proc Natl Acad Sci USA.

[b21] Lee SW, Kim WJ, Choi YK, Song HS, Son MJ, Gelman IH, Kim YJ, Kim KW (2003). SSeCKS regulates angiogenesis and tight junction formation in blood–brain barrier. Nat Med.

[b22] Lee SW, Youn SW, Kim TY, Suh JW, Koh GY, Kwon YW, Chae IH, Park YB, Kim HS (2009). Angiopoietin-1 protects endothelial cells from hypoxia-induced apoptosis via inhibition of phosphatase and tensin homologue deleted from chromosome ten. Korean Circ J.

[b23] Maltepe E, Schmidt JV, Baunoch D, Bradfield CA, Simon MC (1997). Abnormal angiogenesis and responses to glucose and oxygen deprivation in mice lacking the protein ARNT. Nature.

[b24] Maltepe E, Simon MC (1998). Oxygen, genes, and development: an analysis of the role of hypoxic gene regulation during murine vascular development. J Mol Med.

[b25] Mazure NM, Chauvet C, Bois-Joyeux B, Bernard MA, Nacer-Cherif H, Danan JL (2002). Repression of alpha-fetoprotein gene expression under hypoxic conditions in human hepatoma cells: characterization of a negative hypoxia response element that mediates opposite effects of hypoxia inducible factor-1 and c-Myc. Cancer Res.

[b26] Mohyeldin A, Garzon-Muvdi T, Quinones-Hinojosa A (2010). Oxygen in stem cell biology: a critical component of the stem cell niche. Cell Stem Cell.

[b27] Narravula S, Colgan SP (2001). Hypoxia-inducible factor 1-mediated inhibition of peroxisome proliferator-activated receptor alpha expression during hypoxia. J Immunol.

[b28] Ng KM, Lee YK, Chan YC, Lai WH, Fung ML, Li RA, Siu CW, Tse HF (2010). Exogenous expression of HIF-1 alpha promotes cardiac differentiation of embryonic stem cells. J Mol Cell Cardiol.

[b29] Nichols J, Zevnik B, Anastassiadis K, Niwa H, Klewe-Nebenius D, Chambers I, Scholer H, Smith A (1998). Formation of pluripotent stem cells in the mammalian embryo depends on the POU transcription factor Oct4. Cell.

[b30] Niebruegge S, Bauwens CL, Peerani R, Thavandiran N, Masse S, Sevaptisidis E, Nanthakumar K, Woodhouse K, Husain M, Kumacheva E (2009). Generation of human embryonic stem cell-derived mesoderm and cardiac cells using size-specified aggregates in an oxygen-controlled bioreactor. Biotechnol Bioeng.

[b31] Niwa H, Miyazaki J, Smith AG (2000). Quantitative expression of Oct-3/4 defines differentiation, dedifferentiation or self-renewal of ES cells. Nat Genet.

[b32] Nordhoff V, Hubner K, Bauer A, Orlova I, Malapetsa A, Scholer HR (2001). Comparative analysis of human, bovine, and murine Oct-4 upstream promoter sequences. Mamm Genome.

[b33] Nourse MB, Halpin DE, Scatena M, Mortisen DJ, Tulloch NL, Hauch KD, Torok-Storb B, Ratner BD, Pabon L, Murry CE (2010). VEGF induces differentiation of functional endothelium from human embryonic stem cells: implications for tissue engineering. Arterioscler Thromb Vasc Biol.

[b34] Ohlstein B, Kai T, Decotto E, Spradling A (2004). The stem cell niche: theme and variations. Curr Opin Cell Biol.

[b35] Olsson AK, Dimberg A, Kreuger J, Claesson-Welsh L (2006). VEGF receptor signalling—in control of vascular function. Nat Rev Mol Cell Biol.

[b36] Ong LL, Li W, Oldigs JK, Kaminski A, Gerstmayer B, Piechaczek C, Wagner W, Li RK, Ma N, Steinhoff G (2010). Hypoxic/normoxic preconditioning increases endothelial differentiation potential of human bone marrow CD133+ cells. Tissue Eng Part C Methods.

[b37] Powers DE, Millman JR, Huang RB, Colton CK (2008). Effects of oxygen on mouse embryonic stem cell growth, phenotype retention, and cellular energetics. Biotechnol Bioeng.

[b38] Purpura KA, George SH, Dang SM, Choi K, Nagy A, Zandstra PW (2008). Soluble Flt-1 regulates Flk-1 activation to control hematopoietic and endothelial development in an oxygen-responsive manner. Stem Cells.

[b39] Ramirez-Bergeron DL, Runge A, Dahl KD, Fehling HJ, Keller G, Simon MC (2004). Hypoxia affects mesoderm and enhances hemangioblast specification during early development. Development.

[b40] Ryan HE, Lo J, Johnson RS (1998). HIF-1 alpha is required for solid tumor formation and embryonic vascularization. EMBO J.

[b41] Semenza GL (1999). Regulation of mammalian O2 homeostasis by hypoxia-inducible factor 1. Annu Rev Cell Dev Biol.

[b42] Semenza GL (2003). Targeting HIF-1 for cancer therapy. Nat Rev Cancer.

[b43] Tomita S, Ueno M, Sakamoto M, Kitahama Y, Ueki M, Maekawa N, Sakamoto H, Gassmann M, Kageyama R, Ueda N (2003). Defective brain development in mice lacking the Hif-1alpha gene in neural cells. Mol Cell Biol.

[b44] van den Berg DL, Snoek T, Mullin NP, Yates A, Bezstarosti K, Demmers J, Chambers I, Poot RA (2010). An Oct4-centered protein interaction network in embryonic stem cells. Cell Stem Cell.

[b45] Vittet D, Prandini MH, Berthier R, Schweitzer A, Martin-Sisteron H, Uzan G, Dejana E (1996). Embryonic stem cells differentiate in vitro to endothelial cells through successive maturation steps. Blood.

[b46] Wang M, Su Y, Sun H, Wang T, Yan G, Ran X, Wang F, Cheng T, Zou Z (2010). Induced endothelial differentiation of cells from a murine embryonic mesenchymal cell line C3H/10T1/2 by angiogenic factors in vitro. Differentiation.

[b47] Warnecke C, Zaborowska Z, Kurreck J, Erdmann VA, Frei U, Wiesener M, Eckardt KU (2004). Differentiating the functional role of hypoxia-inducible factor (HIF)-1alpha and HIF-2alpha (EPAS-1) by the use of RNA interference: erythropoietin is a HIF-2alpha target gene in Hep3B and Kelly cells. FASEB J.

[b48] Yeo EJ, Chun YS, Cho YS, Kim J, Lee JC, Kim MS, Park JW (2003). YC-1: a potential anticancer drug targeting hypoxia-inducible factor 1. J Natl Cancer Inst.

[b49] Ying QL, Wray J, Nichols J, Batlle-Morera L, Doble B, Woodgett J, Cohen P, Smith A (2008). The ground state of embryonic stem cell self-renewal. Nature.

[b50] Yoshida Y, Takahashi K, Okita K, Ichisaka T, Yamanaka S (2009). Hypoxia enhances the generation of induced pluripotent stem cells. Cell Stem Cell.

