# Supplementary material for: Hypoxic priming of mESCs accelerates vascular-lineage differentiation through HIF1-mediated inverse regulation of Oct4 and VEGF
Source: EMBO Mol Med. 2012 Jul 23;4(9):924–38. doi: 10.1002/emmm.201101107 (PMC3491825; doi:10.1002/emmm.201101107)
Supplement: Supplementary file 1 [file emmm0004-0924-SD1.pdf]

EMBO
Molecular Medicine

Manuscript EMM-2011-01107

Hypoxic priming of mESCs accelerates vascular-lineage differentiation through HIF1-mediated inverse regulation of Oct4 and VEGF

Sae-Won Lee, Han-Kyul Jeong, Ji-Young Lee, Jimin Yang, Eun Ju Lee, Su-Yeon Kim, Seock-Won Youn, Jaewon Lee, Woo Jean Kim, Kyu-Won Kim, Jeong Mook Lim, Jong-Wan Park, Young-Bae Park, Hyo-Soo Kim

Corresponding author: Hyo-Soo Kim, Seoul National University Hospital

Review timeline:

Submission date:	28 November 2011
Accepted:	18 May 2012

Transaction Report:

No Peer Review Process File is available with this article, as the authors have chosen not to make the review process public in this case.
