# Supplementary material for: Hypoxic priming of mESCs accelerates vascular-lineage differentiation through HIF1-mediated inverse regulation of Oct4 and VEGF
Source: EMBO Mol Med. 2012 Jul 23;4(9):924–38. doi: 10.1002/emmm.201101107 (PMC3491825; doi:10.1002/emmm.201101107)

Table of content

Supplementary Figure legends -- 1

Supplementary Table -- 3

Supplementary Figure S1 -- 4

Supplementary Figure S2 -- 5

Supplementary Figure S3 -- 6

Supplementary Figure S4 -- 7

Supplementary Figure S5 -- 8

SUPPLEMENTARY TEXT

Supplementary Figure legends

Supplementary Figure legend of Figure S1. Evaluation of the vascular differentiation potential of hypoxia-primed EBs. The E14 cells were analyzed by FACS analysis for PECAM, VE-cadherin (endothelial cells) and SMA (smooth muscle cells) on day 3 after EB reattachment. Quantitative graph from 4-different experiments. The percentage was normalized to normoxic group and shown as percent fold (n=4, $p<0.05$). N, Normoxic-EBs; H, Hypoxia-primed EBs.

Supplementary Figure legend of Figure S2. The endothelial differentiation of hypoxia-primed EBs is dependent on HIF-1α. (a) EBs was treated with the indicated concentration of YC1, HIF-1α blocker (Sigma), under normoxic or hypoxic conditions. Western blotting of HIF-1α (top). Quantitative graph (bottom) (n=5, *$p<0.05$). **(b)** Hypoxia responsive gene (VEGF) and vascular marker genes (PECAM, VEGFR2, VE-cadherin) were investigated with real-time PCR (n=4, *$p<0.05$).

Supplementary Figure legend of Figure S3. Evaluation of the vascular differentiation potential in HIF-1α knock-down hypoxia-primed EBs. The E14 and shHIF1-E14 cells were analyzed by FACS analysis for PECAM, VE-cadherin (endothelial cells) and SMA (smooth muscle cells) on day 3 after EB reattachment. Nor, Normoxic-EBs; Hy, Hypoxia-primed EBs; shHIF1/Hy, Hypoxia-primed shHIF1 EBs.

Supplementary Figure legend of Figure S4. (a) Gross photographs of mice on post-operation day 3. In the HyEB-injection group, the ischemic limbs (arrow) were comparatively good. In contrast, the NorEB-injection group showed limb loss (arrowhead). **(b)** Quantitative graph of foot length after ischemic surgery (n=5 per group).

Supplementary Figure legend of Figure S5. Vascular differentiation of engrafted cells from hypoxia-primed EBs on post-transplantation day 7. Capillary endothelial cell stained with BS1-lectin (green) and VE-cadherin (white) is co-localized with DiI immunofluorescence (red) in ischemic hindlimb tissues. Magnification=x630, scale bar=20 μm.

Supplementary table S1. Sequence of the primers for Real-time PCR and ChIP assay.

Name		Primer sequence	Size(bp)
Oct4	F	GAAGCCCTCCCTACAGCAGA	297
	R	CAGAGCAGTGACGGGAACAG	
Nanog	F	CCCCACAAGCCTTGGAATTA	255
	R	CTCAAATCCCAGCAACCACA	
VEGF	F	CGGATCAAACCTCACCAAAG	131
	R	TTTCTCCGCTCTGAACAAGG	
Ncam	F	AGATGGTCAGTTGCTGCCAA	187
	R	AGAAGACGGTGTGTCTGCTT	
Nestin	F	TAGAGGTGCAGCAGCTGCAG	170
	R	AGCGATCTGACTCTGTAGAC	
Troma-1	F	ATCGAGATCACCACCTACCG	241
	R	TCTTCACAACCACAGCCTTC	
Desmin	F	TGACAACCTGATAGACGACC	180
	R	TTAAGGAACGCGATCTCCTC	
SMA	F	ACTGGGACGACATGGAAAAG	240
	R	CATCTCCAGAGTCCAGCACA	
VEGFR2	F	TAAGGGCATGGAGTTCTTGG	249
	R	CAGAGCAACACACCGAAAGA	
PECAM	F	TGCAGGAGTCCTTCTCCACT	235
	R	ACGGTTTGATTCCACTTTGC	
VE-cadherin	F	CGTGGTGGAAACACAAGATG	181
	R	TGGGTCCACAACAGTCAGAA	
GAPDH	F	ACTCCACTCACGGCAAATTC	171
	R	TCTCCATGGTGGTGAAGACA	
rHRE1	F	GTTCAGAGCATGGTGTAGGAGCA	-1293 ~
	R	GACACTAAGGAGACGGGATTAGG	-1288
rHRE2	F	CAGAAAACCACTCTAGGGAAGTT	-1176 ~
	R	TCTCCACCTCTCCTCAAAGCAG	-1172
rHRE3	F	CCTTAACTGTGAGGGGATGG	-622 ~
	R	CCAGAGTTTAGAGGCTCTACACC	-618
rHRE4	F	CTCCAGAGGATGGCTGAGTGGGC	-290 ~
	R	TCTGGACAGGACAACCCTTAGGACG	-286

Supplementary Figure S1

[FACS on day 3]

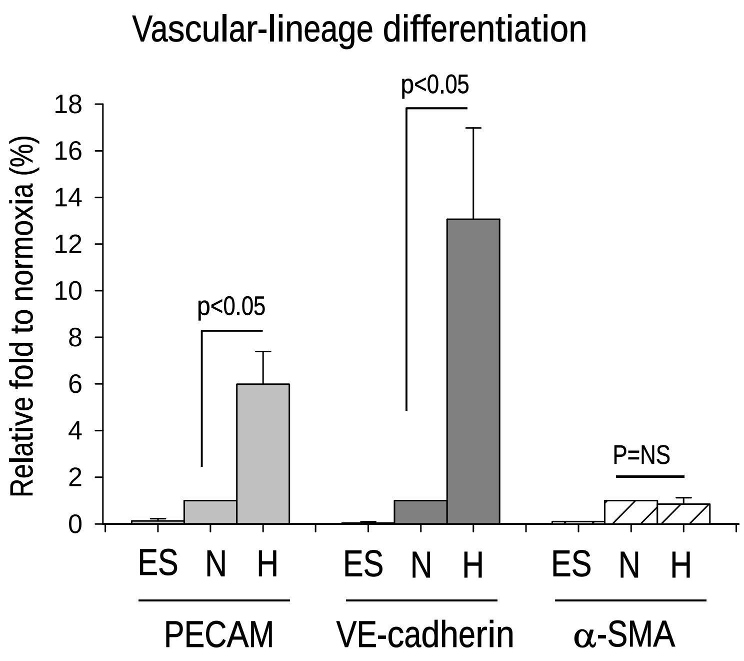

Supplementary Figure S2

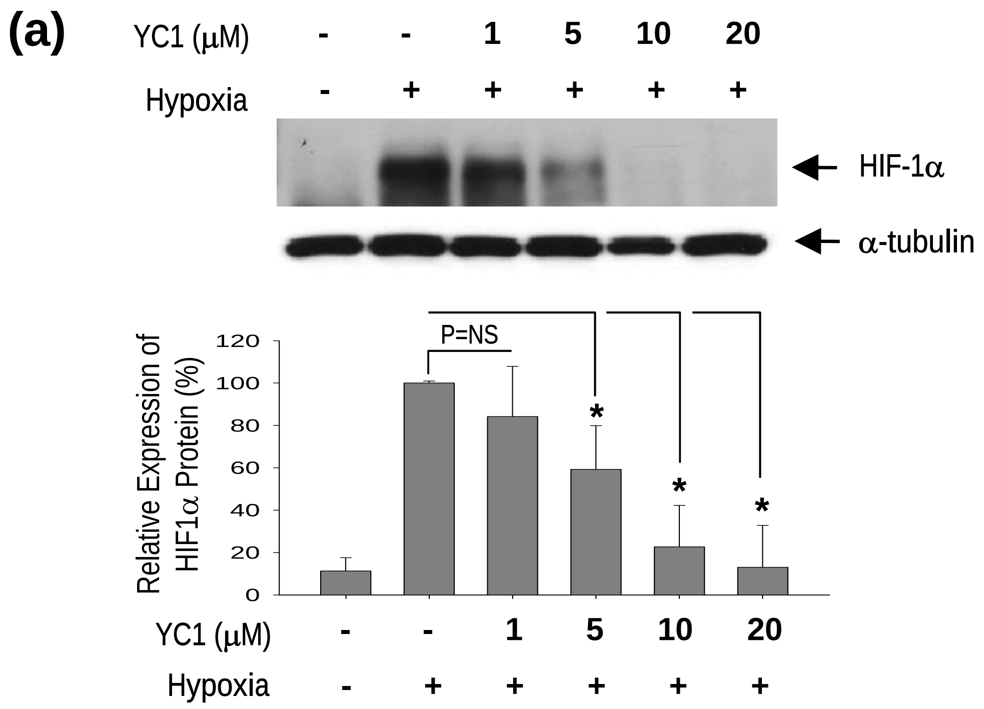

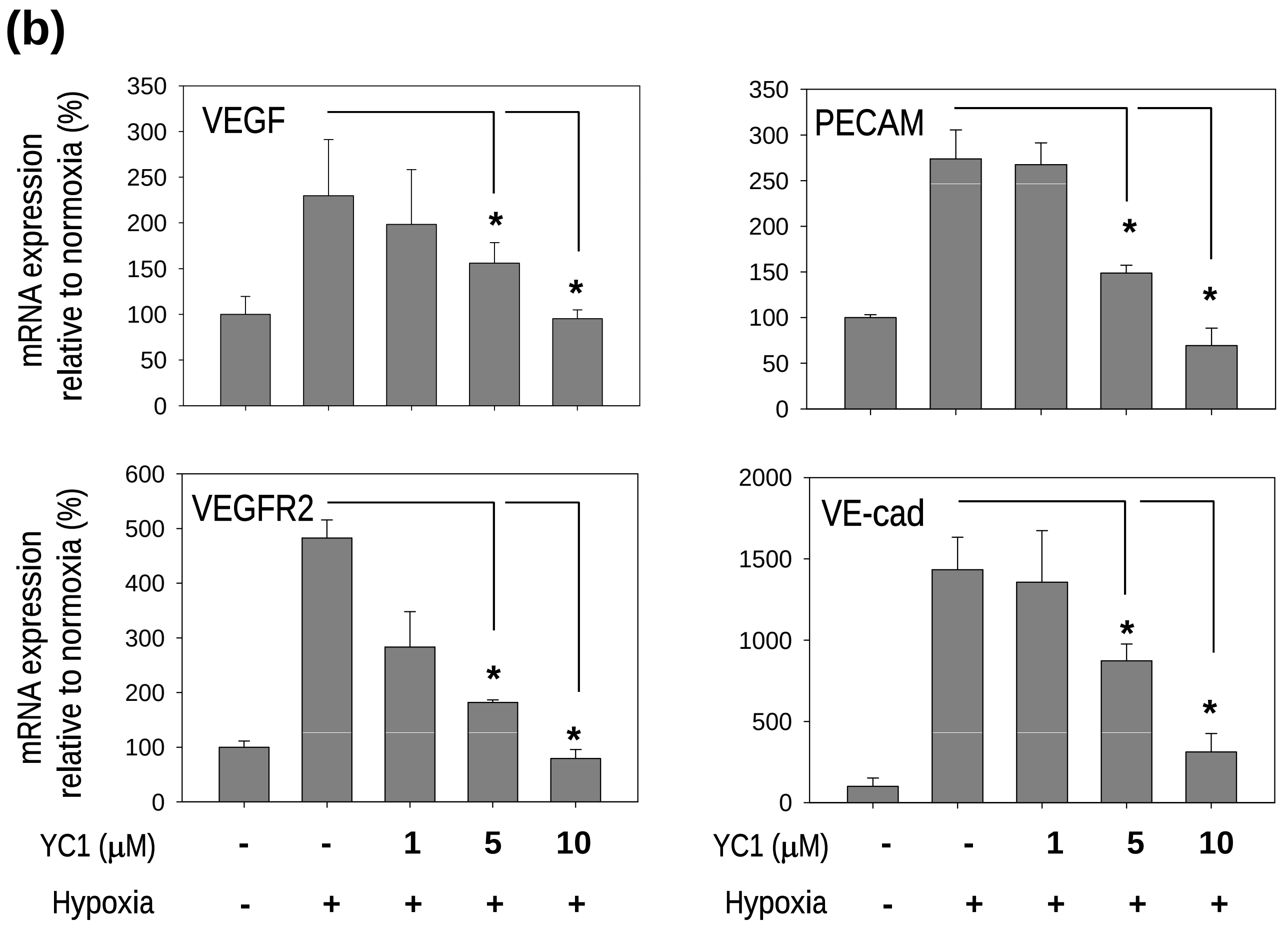

Supplementary Figure S3

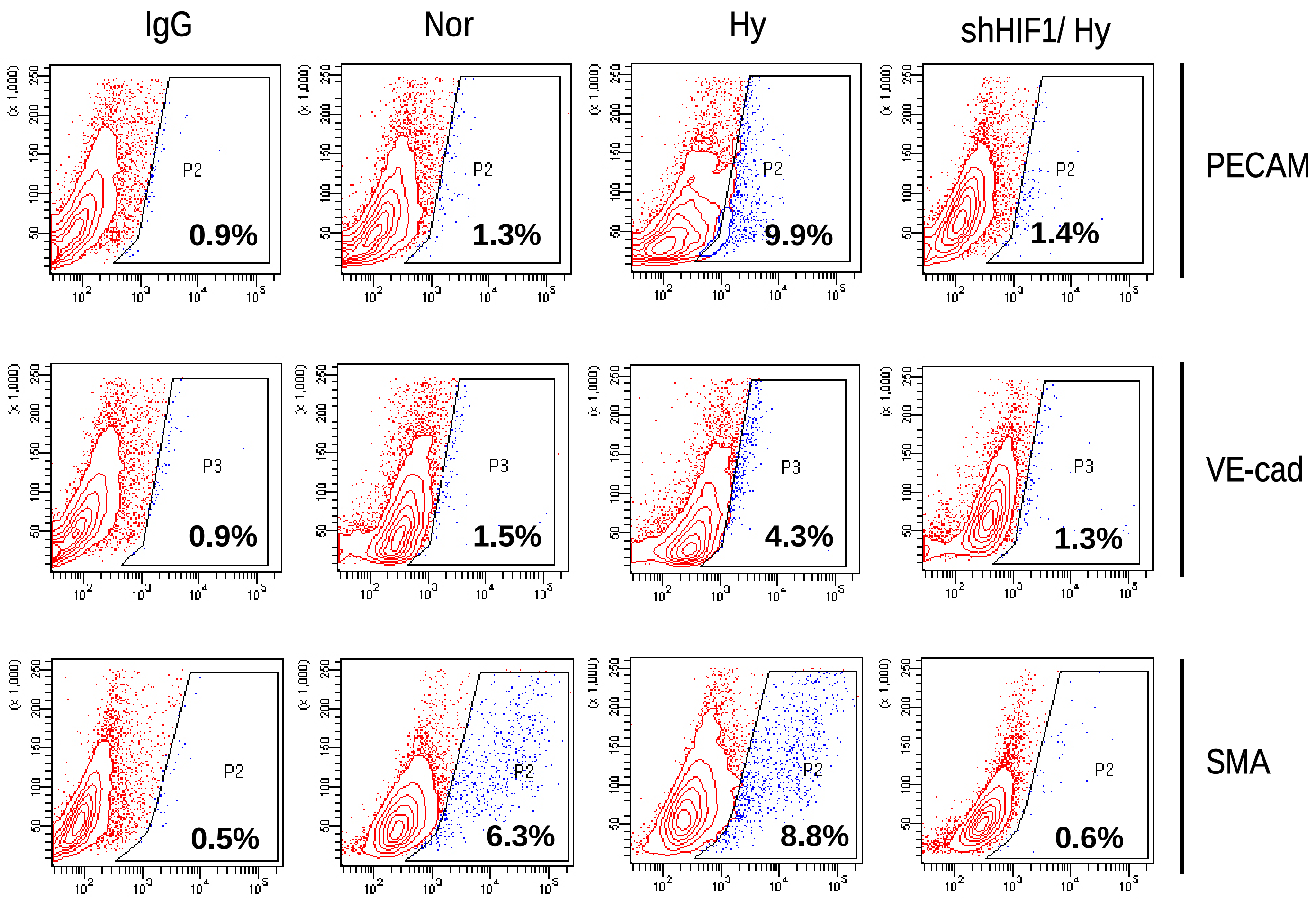

Supplementary Figure S4

(a)

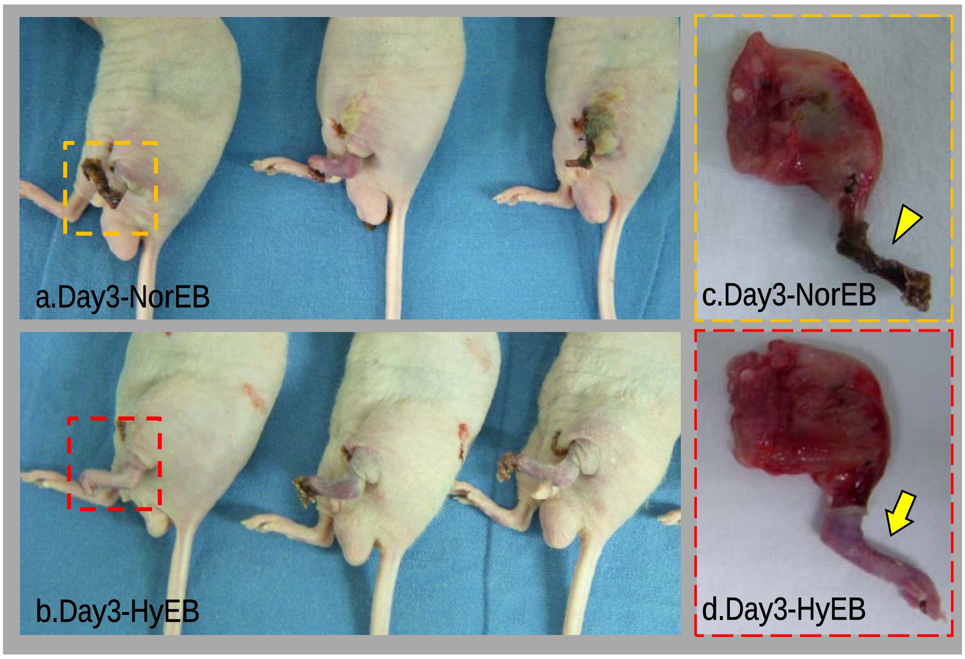

(b)

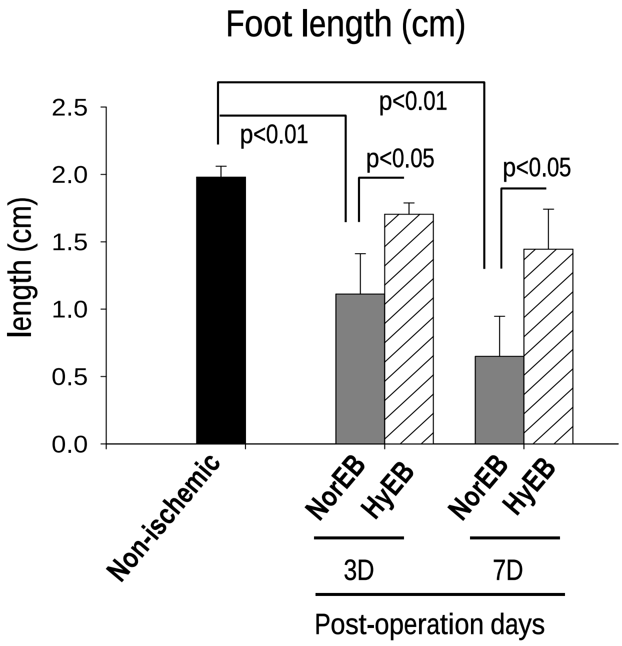

Supplementary Figure S5

BS1-lectin / DiI / VE-cadherin / Nueclus / Phase bright

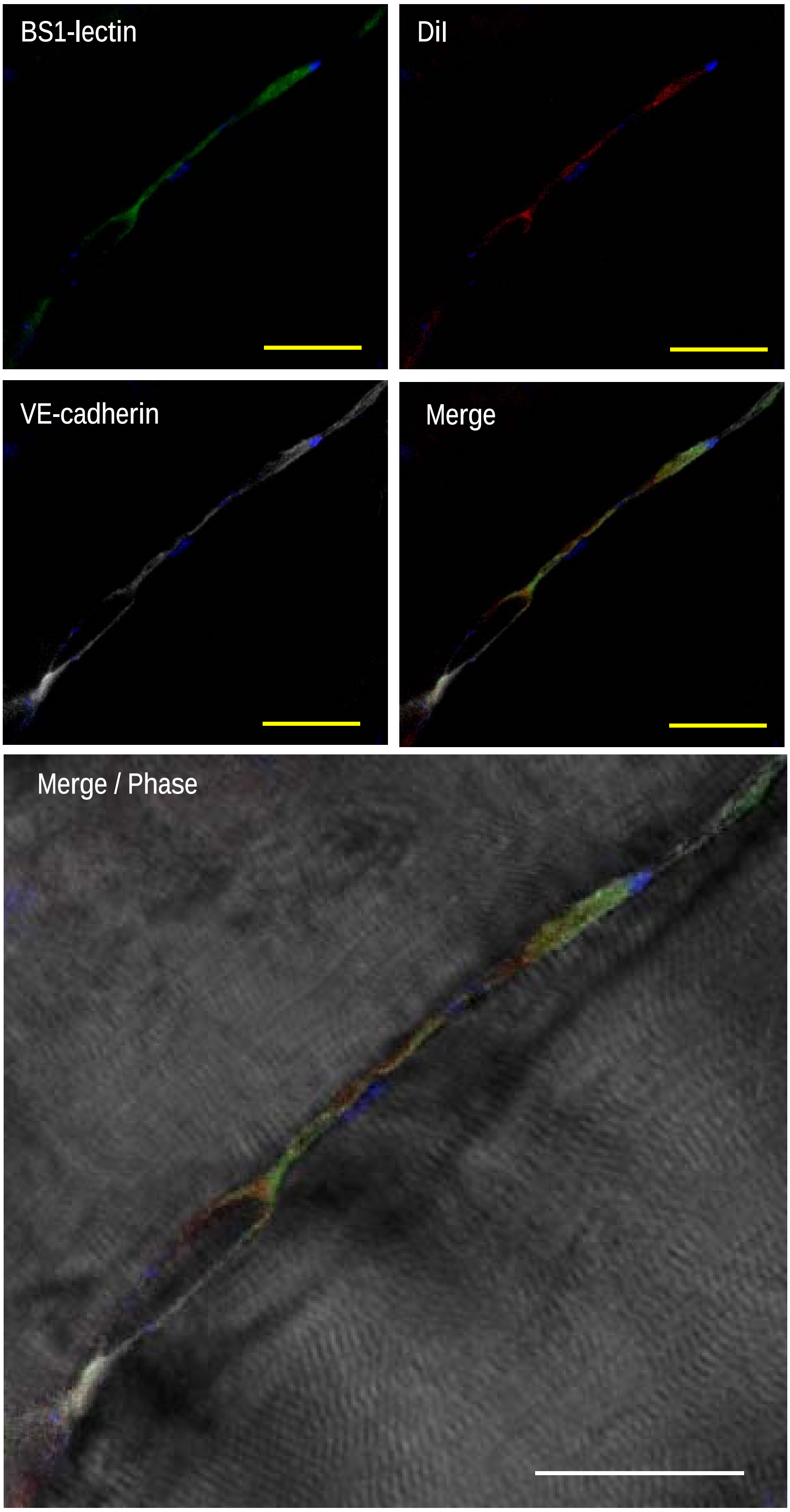

Supplement: Supplementary file 2 [file emmm0004-0924-SD2.pdf]
